# Systematic characterization of all *Toxoplasma gondii* TBC domain-containing proteins identifies an essential regulator of Rab2 in the secretory pathway

**DOI:** 10.1371/journal.pbio.3002634

**Published:** 2024-05-07

**Authors:** Justin J. Quan, Lachezar A. Nikolov, Jihui Sha, James A. Wohlschlegel, Isabelle Coppens, Peter J. Bradley

**Affiliations:** 1 Department of Microbiology, Immunology and Molecular Genetics, University of California, Los Angeles, Los Angeles, California, United States of America; 2 Department of Biology, Indiana University, Bloomington, Indiana, United States of America; 3 Department of Biological Chemistry and Institute of Genomics and Proteomics, University of California, Los Angeles, Los Angeles, California, United States of America; 4 Department of Molecular Microbiology and Immunology, Johns Hopkins University Bloomberg School of Public Health, Baltimore, Maryland, United States of America; 5 Molecular Biology Institute, University of California, Los Angeles, Los Angeles, California, United States of America; Deakin University, Australia, AUSTRALIA

## Abstract

*Toxoplasma gondii* resides in its intracellular niche by employing a series of specialized secretory organelles that play roles in invasion, host cell manipulation, and parasite replication. Rab GTPases are major regulators of the parasite’s secretory traffic that function as nucleotide-dependent molecular switches to control vesicle trafficking. While many of the Rab proteins have been characterized in *T*. *gondii*, precisely how these Rabs are regulated remains poorly understood. To better understand the parasite’s secretory traffic, we investigated the entire family of Tre2-Bub2-Cdc16 (TBC) domain-containing proteins, which are known to be involved in vesicle fusion and secretory protein trafficking. We first determined the localization of all 18 TBC domain-containing proteins to discrete regions of the secretory pathway or other vesicles in the parasite. Second, we use an auxin-inducible degron approach to demonstrate that the protozoan-specific TgTBC9 protein, which localizes to the endoplasmic reticulum (ER), is essential for parasite survival. Knockdown of TgTBC9 results in parasite growth arrest and affects the organization of the ER and mitochondrial morphology. TgTBC9 knockdown also results in the formation of large lipid droplets (LDs) and multi-membranous structures surrounded by ER membranes, further indicating a disruption of ER functions. We show that the conserved dual-finger active site in the TBC domain of the protein is critical for its GTPase-activating protein (GAP) function and that the *Plasmodium falciparum* orthologue of TgTBC9 can rescue the lethal knockdown. We additionally show by immunoprecipitation and yeast 2 hybrid analyses that TgTBC9 preferentially binds Rab2, indicating that the TBC9-Rab2 pair controls ER morphology and vesicular trafficking in the parasite. Together, these studies identify the first essential TBC protein described in any protozoan and provide new insight into intracellular vesicle trafficking in *T*. *gondii*.

## Abstract 2

Toxoplasma gondii regulates vesicle trafficking by secreting Rab GTPases from specialised organelles. This study characterizes all Tre2-Bub2-Cdc16 (TBC) domain-containing proteins in T. gondii, identifying TBC9 as an essential regulator of Rab2 that controls intracellular vesicular trafficking.

## Introduction

*Toxoplasma gondii* is an obligate intracellular parasite in the phylum Apicomplexa that causes the disease toxoplasmosis and infects all mammals including approximately one-third of the human population [1,2]. Although most human infections remain asymptomatic, immunocompromised individuals or congenitally infected neonates are vulnerable to more severe symptoms such as cardiomyopathy, blurred vision, encephalitis, or fatality if left untreated [3]. *T*. *gondii* is the most widespread apicomplexan and serves as a model system for studying other apicomplexan parasites, such as *Cryptosporidium* spp., which causes diarrheal disease, and *Plasmodium falciparum*, which causes malaria [4,5]. In addition to the standard eukaryotic organelles, apicomplexans share a number of unique organelles such as the inner membrane complex (IMC), micronemes, rhoptries, apicoplast, and dense granules that facilitate host cell invasion and intracellular survival [6,7]. As these unique organelles often contain parasite-specific proteins, they are considered excellent targets for therapeutic intervention.

One interesting aspect of *T*. *gondii* is its highly polarized secretory pathway, which delivers secretory proteins to organelles at the apical end of the parasite [8,9]. As in other eukaryotic cells, secretory traffic is initiated by co-translational translocation of protein cargo into the endoplasmic reticulum (ER). In *T*. *gondii*, this traffic then passes through a single Golgi stack which is positioned just anterior to the parasite’s nucleus [7]. After exiting the Golgi, vesicle-based trafficking allows the protein cargo to be directed through post-Golgi compartments to the secretory micronemes, rhoptries, and dense granules, which discharge their contents sequentially for motility, host cell invasion, parasitophorous vacuole (PV) formation, and modification of the PV post-invasion [7,8]. In addition to the secretory organelles, protein cargo from the secretory pathway is also targeted to the IMC, apicoplast, plant-like vacuolar compartment (PLVAC), endosome-like compartment (ELC), or parasite’s surface [7,10–12]. Specific signals have been identified for targeting to most of these compartments, as has some of the vesicle trafficking machinery [10,13]. However, a detailed mechanistic understanding of vesicle sorting as well as the identification of many components of the trafficking machinery has yet to be completed.

Similar to other eukaryotic cells, one of the major regulators of *Toxoplasma* secretory traffic is the Rab family of small GTPases [14]. Rab proteins are tethered to membranes by C-terminal prenylation and function as molecular switches that cycle between an inactive GDP-bound state and an active GTP-bound state to regulate secretory and vesicular traffic [15]. Rabs are regulated by guanine nucleotide exchange factors (GEFs) that promote the exchange of GDP for GTP as well as GTPase activating proteins (GAPs), which hydrolyze GTP to GDP [16]. Twelve Rab proteins have been identified in *T*. *gondii*, which appear to be conserved across most of the eukarya [17,18]. The proteins localize to various secretory or vesicular compartments such as the ER/Golgi, Golgi, post Golgi compartments, or cytoplasmic vesicles [17]. Functional assessment of the Rabs, by dominant negative or overexpression screening, has confirmed their importance in protein trafficking for parasite fitness. For example, overexpression of Rab2, Rab4, Rab5A, Rab5B, and Rab5C dramatically affect parasite growth and overexpression of Rab5A and Rab5C results in mistargeting of rhoptry and microneme cargo into the PV [17,19,20]. Rab11A has been shown to regulate dense granule secretion and Rab11B functions in IMC biogenesis [21,22]. In addition, some of the GEFs that activate Rabs have been described including a Vps9 and a Vps11 domain-containing protein, although their target Rabs have not been identified [23,24].

While several Rab GEFs have been studied in *T*. *gondii*, the GAP proteins responsible for inactivating Rabs have not been explored. Rab GAPs are typically characterized by the presence of a TBC (Tre-2/Bub2/Cdc16) domain and act by increasing the intrinsic GTPase activity of the Rab protein, resulting in a conversion from the GTP to GDP bound form [25]. TBC domain-containing proteins (hereafter termed TBC proteins) often contain other functional domains that presumably contribute to their function in various locations within the cell. The TBC family is poorly studied in most systems as there are typically many family members that often have similar localizations, suggesting functional redundancy (e.g., there are 41 TBC proteins in humans) [25,26]. In addition, there are a small number of other proteins that lack a TBC domain but can still function as Rab GAPs [16,27]. The TBC proteins in most parasites remain largely unstudied, and none have been localized or functionally assessed in *T*. *gondii*. In addition, phylogenetic analyses suggest that the most recent common ancestor of the eukaryotes contained several TBC proteins, which gave rise to a number of TBC clades in different eukaryotic lineages [25]. The relationships among clades are poorly resolved likely due to their ancient divergences, which complicates studies of TBC family proteins as well-defined orthologues are frequently difficult to determine.

Given the gap in our understanding of vesicular transport regulators and GAPs, we performed a systematic analysis of TBC proteins in *T*. *gondii*. In this study, we identify 18 TBC proteins and localize them to discrete compartments of the secretory pathway or other vesicles in the parasite. We then focus on the ER-localized TgTBC9 and demonstrate that conditional knockdown of this family member results in a lethal growth arrest, disruption of the ER, aberrant mitochondrial morphology, and the formation of large lipid droplets (LDs) and ER encapsulated structures. We show by mutagenesis that the GAP activity of TgTBC9 is critical for function and that the *P*. *falciparum* orthologue of TgTBC9 can rescue the *Toxoplasma* knockdown. We also show that TgTBC9 directly binds to the ER-Golgi localized Rab2 protein, indicating this is the major target of TgTBC9 [17]. This work substantially builds on our understanding of vesicular protein targeting and identifies the first essential TBC protein identified in any protozoan, thus revealing a critical player in secretory traffic in *T*. *gondii* and related apicomplexan parasites.

## Results

### *T*. *gondii* encodes 18 TBC proteins that are mostly unique to the Apicomplexa

**Fig 1:**
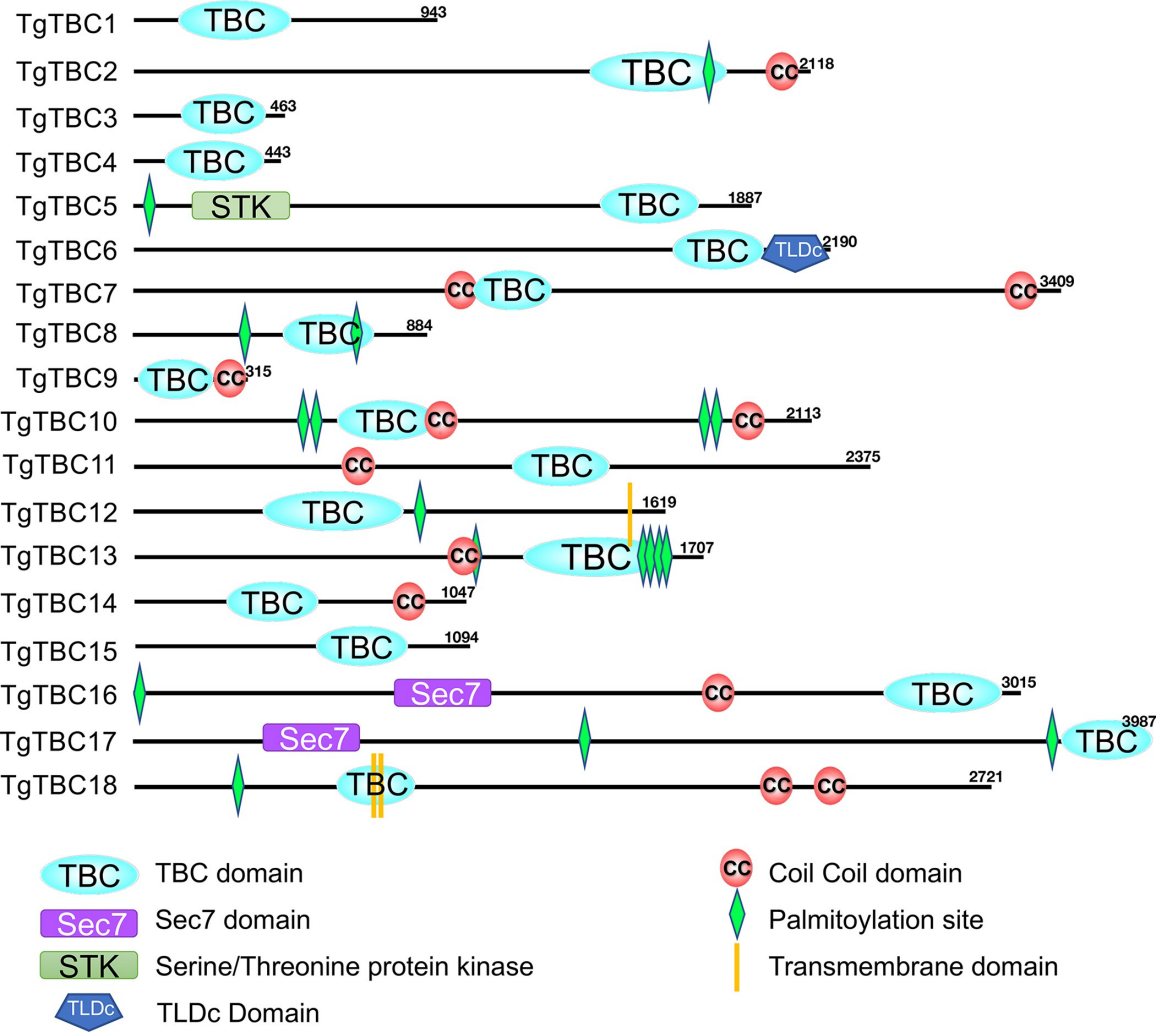
*Toxoplasma gondii* contains 18 TBC proteins. Diagram showing the domains present in the *Toxoplasma* TBC proteins. The approximate position of the TBC and other domains as predicted by SMART, PFAM, and NCBI conserved protein domain search tools are shown [28–30]. TBC, Tre2–Bub2–Cdc16 (TBC) domain-containing proteins; Sec7, Sec7-domain-containing; STK, Serine/Threonine protein kinase; TLDc, TBC, LysM, Domain Catalytic domain; CC, coil-coil domain; TM, transmembrane; SMART, Simple Modular Architecture Research Tool.

To identify the repertoire of TBC proteins in *T*. *gondii*, we searched for proteins that contain a TBC domain as predicted by NCBI, InterPro, and SMART conserved domain searches [28–30]. This reveals a total of 18 TBC proteins which range from a molecular weight of 35 kDa to 426 kDa (Fig 1 and Table 1). Nine of these are over 200 kDa in size, which is considerably larger than most TBC proteins (e.g., human TBC proteins range from 34.97 kDa (TBC1D21) to 158.69 kDa (UPS6)) [16]. Similar to that seen in other systems, the TBC domain is situated at various positions in the proteins, and they frequently contain additional identifiable protein motifs such as coiled-coil (CC) domains, transmembrane domains (TM), kinase domains, or Sec7 catalytic domains (Fig 1). The Sec7 domain is a well-characterized motif belonging to the GEF family, which are known to activate Rabs [31]. As TgTBC15 and TgTBC16 contain both a Sec7 domain and TBC domain, they harbor both positive and negative regulators of Rab proteins (Fig 1). Members of this novel subfamily of the TBC proteins that contain both TBC and Sec7 domains are often called TBC-Sec7 (TBS) proteins and are only found in the protists [32].

**Table 1:** Summary of 18 TBC domain-containing proteins in *Toxoplasma gondii*.

Gene name	ToxoDB-ID	Protein size (kDa)^a^	Phenotype score^b^	Localization (tachyzoite)	IxxDxxR predicted active site	YxQ predicted active site
TgTBC1	TgGT1_275350	102	−0.58	Golgi	Yes	Yes
TgTBC2	TgGT1_274130	226	−1.84	Golgi	No	Yes
TgTBC3	TgGT1_218870	52	−0.74	Golgi	Yes	Yes
TgTBC4	TgGT1_285730	49	−0.36	Not expressed	Yes	Yes
TgTBC5	TgGT1_250680	207	−0.06	Cytoplasmic vesicles	No	No
TgTBC6	TgGT1_237280	233	−0.16	ER	No	No
TgTBC7	TgGT1_216430	358	0.09	Not expressed	Yes	Yes
TgTBC8	TgGT1_261200	99	−1	IMC	Yes	Yes
TgTBC9	TgGT1_226550	35	−4.82	ER	Yes	Yes
TgTBC10	TgGT1_203910	228	0.53	Cytosolic	Yes	Yes
TgTBC11	TgGT1_239830	257	−2.83	Daughter	No	Yes
TgTBC12	TgGT1_223640	178	−0.6	Cytoplasmic vesicles	Yes	Yes
TgTBC13	TgGT1_221710	182	−2.04	Cytoplasmic vesicles	No	Yes
TgTBC14	TgGT1_289820	114	−1.14	ER	Yes	Yes
TgTBC15	TgGT1_226850	117	0.54	Daughter	Yes	Yes
TgTBC16	TgGT1_312300	324	−1.19	Cytoplasmic vesicles	No	No
TgTBC17	TgGT1_266830	426	0.3	Cytoplasmic vesicles	No	Yes
TgTBC18	TgGT1_213325	293	−2.86	Golgi	Yes	Yes

The 18 TBC proteins identified in *T*. *gondii* with their designated gene name, ToxoDB-ID, protein size, localization determined by epitope tagging, and whether or not the dual-finger active site (IxxDxxR or YxQ motifs) are present.

^a^Predicted molecular weight of open reading frame without posttranslational modifications and epitope tagging.

^b^GWCS [36]. A negative score indicates impaired fitness.

ER, endoplasmic reticulum; GWCS, genome-wide CRIPSR screen; IMC, inner membrane complex; TBC, Tre2-Bub2-Cdc16.

Because standard phylogenetic analyses of TBC proteins have been shown to provide limited information, we used the OrthoMCL database to identify orthologues that could be clearly determined (S1A Fig and S1 Table) [33]. We also used BLAST analysis to identify several likely orthologues that were not predicted by OrthoMCL [34]. This showed that TgTBC1-8 have orthologues that are present in a broad range of eukaryotic lineages, suggesting these proteins may carry out functions in common to their eukaryotic counterparts. In contrast, TgTBC9-18 appear to be restricted to protozoans, with TgTBC13-18 being unique to *Toxoplasma* and its closest relatives (e.g., *Neospora caninum*, but not other coccidians or apicomplexans). We also found that 10 of the *T*. *gondii* TBC proteins contain a recognizable dual-finger active site consisting of an “arginine finger” (IxxDxxR) and a “glutamine finger” (YxQ), suggesting TBC domain catalytic activity (S1B Fig and Table 1) [35].

### *Toxoplasma* TBC proteins localize to the secretory pathway and cytoplasmic vesicles

**Fig 2:**
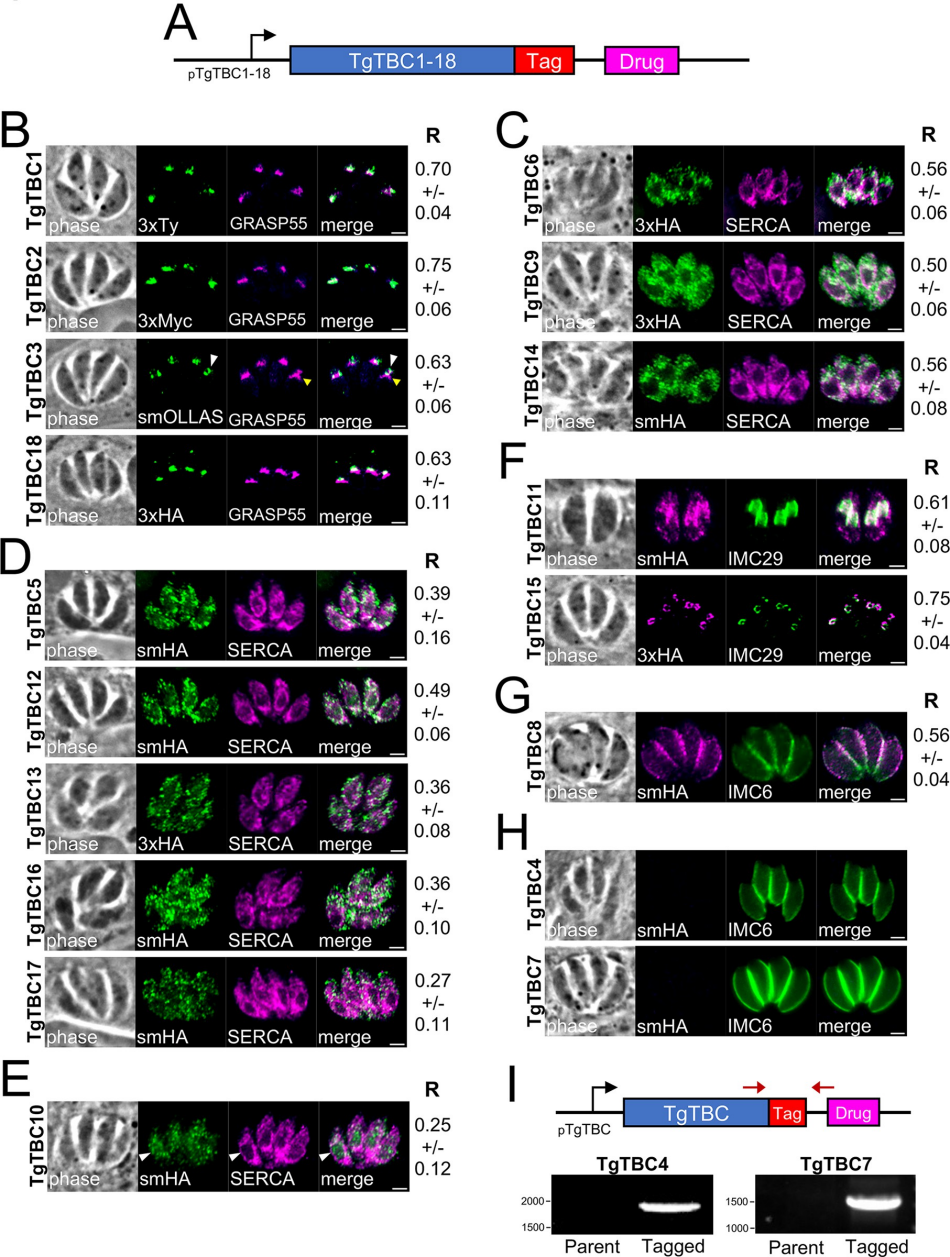
*T. gondii* TBC proteins localize to discrete regions of the secretory pathway and cytoplasmic vesicles. IFAs of endogenously epitope tagged TgTBC1-18 parasites. (A) Diagram of TgTBC1-18 showing the epitope tag and selectable marker. (B) IFA of endogenously tagged TgTBC1, 2, 3, and 18 stained with antibodies against epitope tags and colocalized with GRASP55. The yellow and white arrowheads in the bottom 2 panels denote subtle differences between the TBC protein and GRASP55. Green = endogenously tagged TBC proteins, Magenta = GRASP55-mCherry. Quantification of all colocalizations were quantified by calculating the Pearson’s correlation coefficient (R). Mean values and respective standard deviation of 10–15 parasites are indicated next to the respective image (see also S1 Data). (C) IFA of TgTBC6, 9, and 14 stained with anti-HA and colocalized with SERCA. Green = rabbit anti-HA, Magenta = mouse anti-SERCA. (D) IFA of TgTBC 5, 12, 13, 16, and 17 shown partially colocalizing with SERCA. Green = rabbit anti-HA, Magenta = mouse anti-SERCA. (E) IFA of TgTBC10 shows cytoplasmic and nuclear staining with partial colocalization with SERCA. The white arrowheads show that TgTBC10 is not nuclear excluded. Green = rabbit anti-HA, Magenta = mouse anti-SERCA. (F) IFA of TgTBC11 and TgTBC15 colocalizing with endogenously tagged IMC29^3xV5^ in daughter buds. Magenta = mouse anti-HA, Green = rabbit anti-V5. (G) IFA of TgTBC8 shows staining central portion of the maternal IMC as accessed by IMC6 staining. The white arrowheads indicate basal portions of the parasite where TgTBC8 is absent. Magenta = mouse anti-HA, Green = rabbit anti-IMC6. (H) IFA of TgTBC4 and TgTBC7 with no detectable smHA staining. Magenta = mouse anti-HA, Green = rabbit anti-IMC6. Scale bars for all images, 2 μm. (I) Diagram and PCR of endogenous TgTBC4 and TgTBC7 tagged parasites. Primers labeled as red arrows were used to test gDNA from parental and tagged strains. IFA, indirect immunofluorescence assay; IMC, inner membrane complex; smHA, spaghetti monster HA; TBC, Tre2-Bub2-Cdc16.

To determine the localization of TBC proteins in *T*. *gondii*, we endogenously tagged the C-terminus of each TBC protein with an epitope tag in tachyzoites (Fig 2A) [37]. Proteins with higher expression levels were tagged with a 3xHA, 3xTy, or 3xMyc tag, while those that were likely to have low levels of expression were tagged with either a spaghetti monster HA (smHA) or spaghetti monster OLLAS (smOLLAS) epitope tag [37,38]. Using this approach, we found that TgTBC1, 2, 3, and 18 localizes to a bar-like shape that is positioned anterior to the nucleus, which suggests localization to the Golgi or Golgi-adjacent compartments. We co-stained these 4 proteins with the *cis*-Golgi marker GRASP55 and observed varying degrees of overlap as assessed by Pearson’s correlation coefficients (Fig 2B) [39]. TgTBC1 and 2 colocalize best with GRASP55, while TgTBC3 and 18 appear slightly more posterior to GRASP55, indicating that these proteins localize to trans-Golgi or post-Golgi compartments [39]. Eight of the TBC proteins (TgTBC5, 6, 9, 12, 13, 14, 16, and 17) showed a nuclear-excluded, reticular or spotted cytoplasmic staining indicative of the ER or cytoplasmic vesicles. We co-stained these with the ER marker SERCA which showed that TgTBC6, 9, and 14 colocalized best and were most likely ER-resident proteins (Fig 2C) [40,41]. The remaining 5 were denoted as cytoplasmic vesicles (Fig 2D), some of which likely represent different levels of intersection of the endocytic and exocytic pathways downstream of the Golgi [13], although localization to other types of cytoplasmic vesicles is also possible. TgTBC10 localizes to spots in the cytoplasm, but it is not nuclear-excluded as assessed by SERCA co-staining (Fig 2E).

In addition, three of the TBC proteins were found to localize to the parasite-specific IMC. Two of these, TgTBC11 and TgTBC15, localize to the daughter buds of IMC during endodyogeny, as assessed by colocalization with the early daughter bud marker IMC29 (Fig 2F) [42]. In contrast, TgTBC8 appears to localize to the maternal IMC, as it is peripheral but not present in the apical cap or basal portion of the organelle (Fig 2G) [43]. Finally, in agreement with the low expression levels reported in ToxoDB, TgTBC4 and 7 could not be detected by IFA even though integration of the tag was confirmed by PCR, indicating that these family members are not significantly expressed in the parasite’s tachyzoite stage (Fig 2H and 2I) [44,45].

### TgTBC9 is essential for parasite survival and organization of the ER and mitochondria

**Fig 3:**
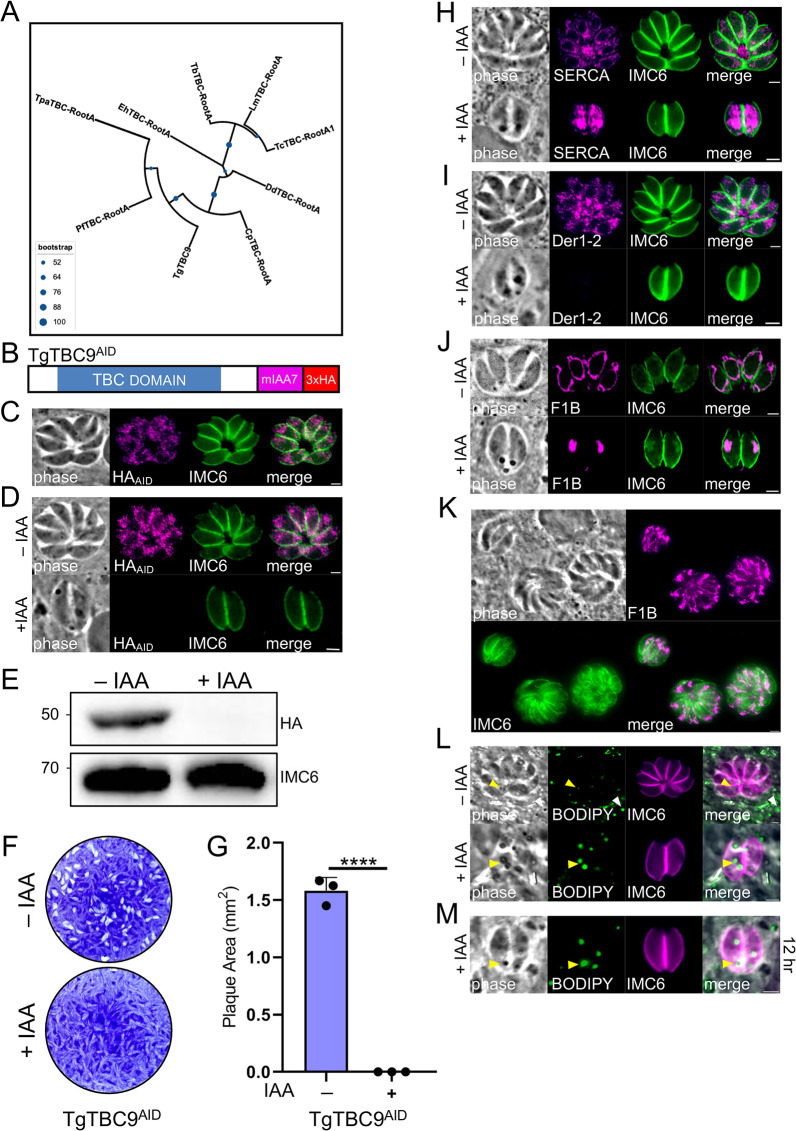
TgTBC9 is essential for parasite survival and organization of the ER and mitochondria. (A) Maximum likelihood tree based on the TBC domains of TgTBC9 and its orthologs from *Plasmodium falciparum* (Pf), *Trypanosoma brucei* (Tb), *Entamoeba histolytica* (Eh), *Cryptosporidium parvum* (Cp), *Theileria parva* (Tp), *Trypanosoma cruzi* (Tc), *Leishmania major* (Lm), *Dictyostelium discoideum* (Dd). Blue spheres denote level of support (1,000 bootstrap replicates). (B) Diagram of TgTBC9^AID^ showing its TBC domain and a mIAA7-3xHA degron tag added to the C-terminus of the protein. (C) IFA of TgTBC9^AID^ localizing to an ER-like pattern. Magenta, mouse anti-HA; green, rabbit anti-IMC6. (D) IFA of TgTBC9^AID^ without (-) or with (+) IAA for 24 h (following a 4 h pretreatment ±IAA) showing that TgTBC9^AID^ is efficiently degraded, resulting in parasite growth arrest. Magenta, mouse anti-HA; green, rabbit anti-IMC6. (E) Western blot analysis of showing TgTBC9^AID^ is efficiently degraded upon IAA treatment. IMC6 is used as a load control. (F) Plaque assays showing that TgTBC9-depleted parasites are unable to form plaques. (G) Quantification of plaque assays at day 7 showing no plaque formation by TgTBC9^AID^ parasites +IAA. All raw data in S1 Data. (H) IFA of TgTBC9^AID^ parasites grown in ±IAA as described in D showing affected ER morphology. Magenta, mouse anti-SERCA; green, rabbit anti-IMC6. (I) TgTBC9^AID-3xTy^ parasites grown in ±IAA as described in D but with staining for Der1-2 using an endogenously tagged Der1-2^3xHA^ strain. Magenta, mouse anti-HA; green, rabbit anti-IMC6. (J) TgTBC9^AID^ parasites grown in ±IAA as described in D but with staining for mitochondrion using anti-F1β ATPase. Magenta, mouse anti-F1β ATPase; green, rabbit anti-IMC6. (K) IFA of TgTBC9^AID^ grown with +IAA for 24 h and then washed and incubated with -IAA for another 24 h (following a 4 h pretreatment +IAA) showing that TgTBC9 deficient parasites resume growth in the absence of IAA. Magenta, mouse anti-F1β ATPase; green, rabbit anti-IMC6. (L) TgTBC9^AID^ parasites grown in ±IAA as described in D but with staining for lipid structures using BODIPY 493/503. Magenta, rabbit anti-IMC6; green, BODIPY 493/503. The white arrowheads denote host lipid structures, while the yellow arrowheads denote parasite lipid structures. (M) TgTBC9^AID^ parasites grown in +IAA for 12 h (following a 4 h pretreatment) staining for lipid structures using BODIPY 493/503. Magenta, rabbit anti-IMC6; green, BODIPY 493/503. The yellow arrowheads denote parasite lipid structures. (****, *P <* 0.0001). Scale bars for all images, 2 μm. ER, endoplasmic reticulum; IFA, indirect immunofluorescence assay; TBC, Tre2-Bub2-Cdc16.

We focused on TgTBC9 because it was assigned the lowest phenotype score (−4.82) of the TBC proteins in the *Toxoplasma* genome-wide CRIPSR screen (GWCS), suggesting that the protein is either important for parasite fitness or essential (Table 1) [36]. Phylogenetic analysis based on the conserved TBC domain of homologs from apicomplexans, amebozoans, and trypanosomatids demonstrates that TgTBC9 is a member of a protozoan-restricted clade of TBC proteins previously denoted as TBC-RootA (S1A Fig) [25]. Neighbor joining analysis shows that TgTBC9 forms a well-supported clade with TBC-RootA homologs from other apicomplexans (*P*. *falciparum*, *T*. *parva*, *C*. *parvum*) (Fig 3A). To determine the function of TgTBC9, we used a conditional knockdown approach by generating parasites with TgTBC9 endogenously tagged with the mIAA7 auxin-inducible degron (AID) fused to 3xHA (TgTBC9^AID^) (Fig 3B). The mIAA7 degron tag was chosen as it is known to be less susceptible to basal degradation in the absence of auxin (3-indoleacetic acid, IAA) [46]. The TgTBC9^AID^ fusion protein localized to an ER-like pattern similar to that seen for the 3xHA tagged version (Figs 2C and 3C). To assess the effects of the TgTBC9 knockdown on parasite growth, intracellular parasites were treated with and without IAA for 4 h, syringe-lysed, and then allowed to infect confluent monolayers for 24 h with and without IAA. Using this approach, we observed that TgTBC9 protein levels decreased to an undetectable level by IFA and western blotting, demonstrating an effective depletion of the protein (Fig 3D and 3E). Replication of the knockdown parasites appears to cease, often arresting at 2 parasites per vacuole, although the overall morphology of the arrested parasites is not grossly affected as assessed by IMC6 staining (Fig 3D). To evaluate the knockdown at a longer time point, we conducted plaque assays for 7 days and found that TgTBC9^AID^ parasites were unable to form plaques upon IAA treatment, demonstrating that TgTBC9 is essential for parasite survival in vitro (Fig 3D and 3G).

Since the TgTBC9 knockdown parasites arrest, we examined a series of organelles in the knockdown strain, including the ER, Golgi, post-Golgi compartment labeled by DrpB, PLVAC, ELC, micronemes, rhoptries, dense granules, IMC, centrosome, apicoplast, and mitochondria. Consistent with the degron tagged protein localizing to the ER, we found TgTBC9 knockdown causes the ER to lose its reticular pattern and become more dense in the cytoplasm as assessed by staining with the ER marker SERCA (Fig 3H) [40]. We also examined the ER marker Der1-2 and found that it was largely lost upon knockdown, again indicating that the ER is affected upon the loss of TgTBC9 (Fig 3I) [47]. Interestingly, the Golgi apparatus, post-Golgi compartment, rhoptries, micronemes, dense granules, PLVAC, ELC, apicoplast, centrosome, and IMC all appear to remain unaffected (S2 Fig). However, we also observed that TgTBC9 knockdown affected the morphology of the mitochondria, which collapsed from its typical lasso-like shape into a single rounded organelle (Fig 3J). This may suggest the parasite is losing viability, but a similar collapse of the mitochondria is seen when the mitochondria-IMC membrane tether is lost without leading to parasite death [48,49]. To determine if the parasite growth arrest was reversible, we removed the auxin following a 24-h knockdown and found that the parasites were able to resume growth (Fig 3K). These data indicate that TgTBC9 is essential for proper ER and mitochondrial morphology and that these effects can be reversed if the knockdown is released before the effects become lethal.

In addition to the organelles described above, we noticed the presence of dense spots in the cytoplasm of TgTBC-depleted parasites by phase contrast microscopy, suggestive of lipid dense structures (Fig 3L). To test this, we stained TgTBC9^AID^ parasites -/+ IAA with BODIPY 493/503, a lipophilic dye that detects stores of neutral lipids. Without IAA, BODIPY stained small spots reminiscent of LDs in both the host cells and parasites as previously described [50]. However, IAA treatment results in large BODIPY-positive structures that correspond to the spots observed by phase contrast microscopy. While we could not see dramatic morphological changes in the ER at earlier time points, the BODIPY-positive spots could readily be detected at 12 h following TgTBC9 knockdown (Fig 3M). As LD droplet biogenesis takes place in the ER, this suggests that ER functions are affected prior to the rounding of the mitochondria [51]. Together, these results indicate that loss of TgTBC9 results in the dysregulation of lipid homeostasis which may result in lipotoxicity that could contribute to parasite death [52,53].

### Ultrastructural analysis of TgTBC9 knockdown parasites

**Fig 4:**
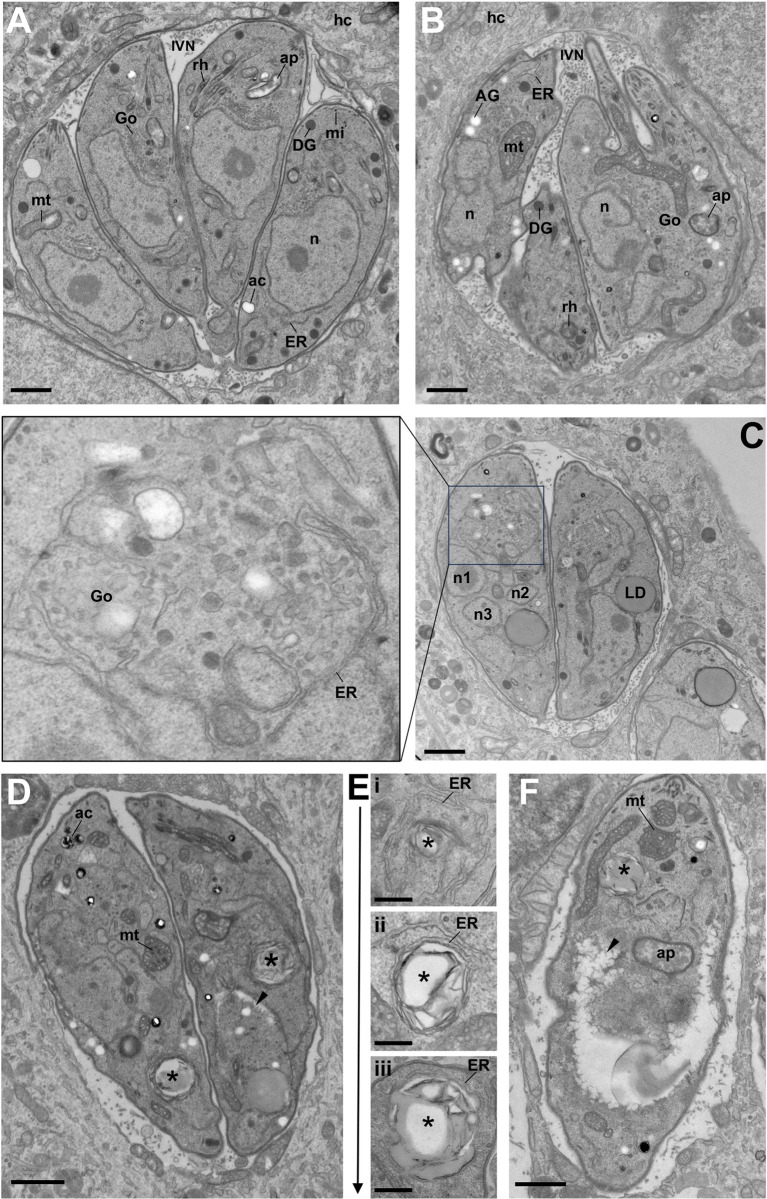
Ultrastructural analysis of TgTBC9 knockdown parasites. (A) TEM of intracellular *Toxoplasma* in HFF for 24 h in the absence of IAA illustrating 4 control (untreated) parasites in a symmetrical organization inside the PV. (B–F) TEM of intracellular *Toxoplasma* in the presence of IAA. (B) Image showing an example of a PV containing 2 or 3 IAA-treated parasites with disorganized parasite morphology. The TgTBC9 knockdown parasites contain aberrant ER structures, a rounded mitochondrion (mt), and the presence of amylopectin granules (AG). (C) Image showing that TgTBC9 knockdown parasites also contain multiple nuclear profiles within one parasite (n1-3), very large LDs, and a disrupted ER-Golgi (Go) connection with accumulated vesicles of various size and electron density. (D, E) Images showing membranous structures surrounded by ER elements (asterisks) with E illustrating the likely progressive steps of their formation/compaction. (F) Parasites showing cytoplasmic clefts in the cytoplasm (arrowheads in D, F), likely preceding parasite death. Asterisks highlights an ER enveloped structure. Ac, acidocalcisome; ap, apicoplast; DG, dense granule; hc, host cell; IVN, intravacuolar network; mi, microneme; n, nucleus; rh, rhoptry. Scales bar for all images, 500 nm. ER, endoplasmic reticulum; HFF, human foreskin fibroblast; LD, lipid droplet; PV, parasitophorous vacuole; TEM, transmission electron microscopy.

To further scrutinize the TgTBC9 depletion phenotype, we examined the knockdown parasites using transmission electron microscopy (TEM, Fig 4). In agreement with our IFA analysis, we found aberrant ER structures, swollen mitochondria with anormal electron-dense cristae, and very large LDs (Fig 4A–4C). Other organelles such as the Golgi, apicoplast, rhoptries, acidocalcisomes, dense granules, and intravacuolar network appear unaffected [5,54,55]. We also observed parasites with varying degrees of morphological defects in the shape of the parasite body (Figs 4B and S3A). In addition, we observed amylopectin granules, which usually typifies the parasite cyst forms, suggesting a state of stress (Fig 4B) [56]. In some instances, we noticed several nuclear profiles, suggestive of asynchronous parasite replication (Fig 4C). Most strikingly, knockdown of TgTBC9 resulted in the accumulation of large osmiophilic structures (up to 1 micron in diameter) surrounded by multiple layers of membranes including ER elements (Fig 4D and 4E). These multi-membranous structures that morphologically differ from LD may also be detected by BODIPY staining (Figs 4D, asterisks and S3B). The TEM analysis suggests a progression of these ER encapsulated structures that increase in size and membrane envelopment (Fig 4E, from panel i to iii), although this cannot be definitively determined. Lastly, we also observed clefts in the cytoplasm that appear to originate near the nuclear envelope, events which likely precede parasite death (Figs 4F and S3C, arrowheads). Together, the ultrastructural analysis shows a disruption of the ER and mitochondria, and suggests a dysregulation of ER-based activities such as lipid droplet formation, lipolysis, and autophagy.

### TBC dual-finger active sites are required for TgTBC9 GAP activity

**Fig 5:**
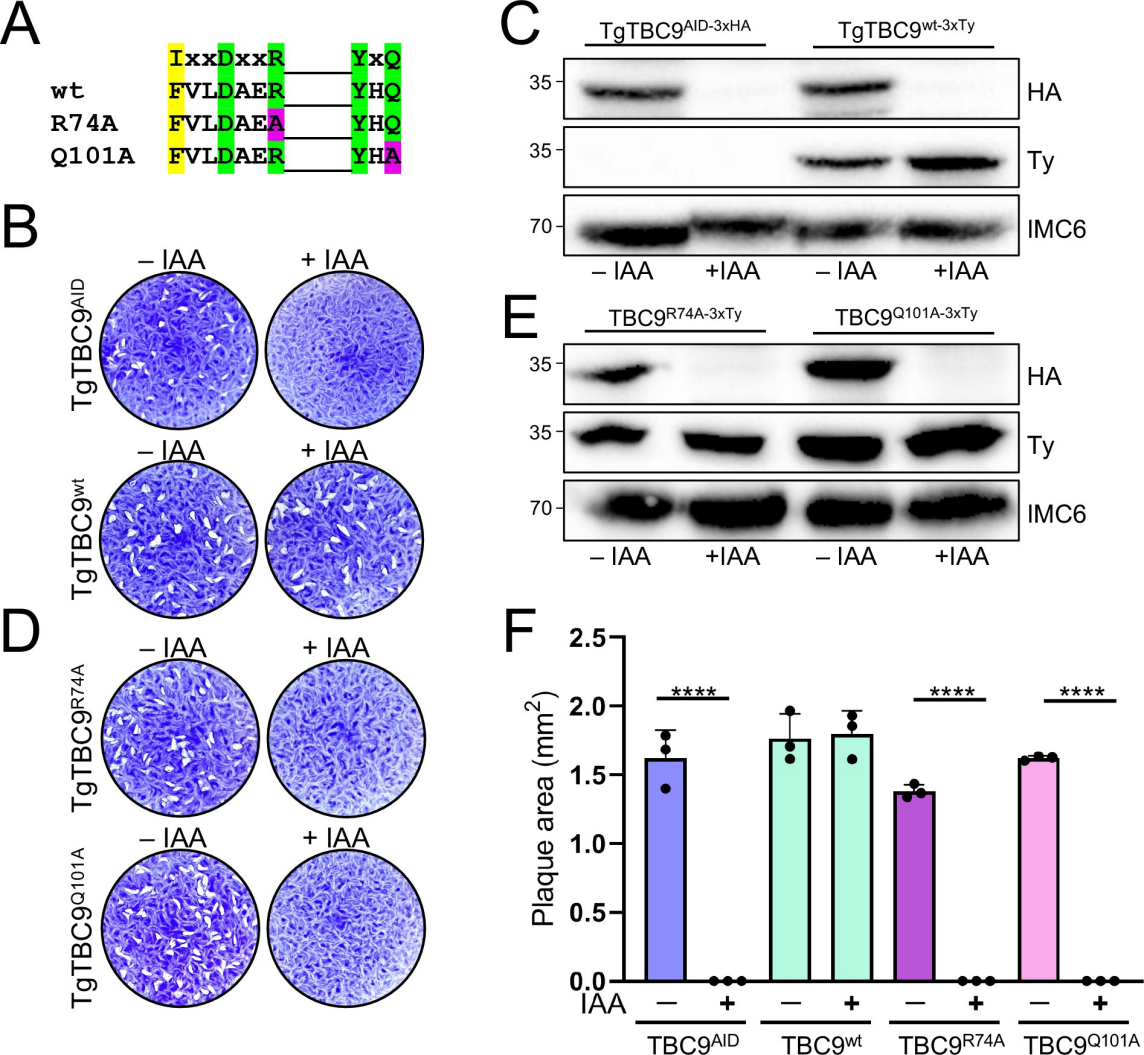
TBC dual-finger active sites are required for TgTBC9 GAP activity. (A) Diagram of the dual-finger consensus and highlighting which TgTBC9 residues in the arginine finger and glutamine finger motifs were mutated to alanine. Green boxes depict strictly conserved residues; yellow boxes depict semi-conserved residues; magenta boxes depict residues that were mutated to alanine. (B) Plaque assays showing that complementation with full-length TgTBC9 restores ability to form plaques upon depletion of endogenous TgTBC9. (C) Western blot analysis showing knockdown of endogenous TgTBC9 and complementation with a Ty-tagged TgTBC9^wt^ copy targeted to the UPRT locus. IMC6 is used as a load control. (D) Plaque assays showing complementation with the TgTBC9^R74A^ or TgTBC9^Q101A^ mutants are unable to rescue the TgTBC9^AID^ knockdown. (E) Western blot analysis showing that the TgTBC9^wt^ and TgTBC9^R74A^ and TgTBC9^Q101A^ mutant parasites have similar levels of expression. IMC6 is used as a load control. (F) Quantification of plaque assays at day 7 showing rescue with TgTBC9^wt^ but no plaque formation by TgTBC9^AID^, TgTBC9^R74A^, and TgTBC9^Q101A^ mutant parasites +IAA (****, *P* ≤ 0.0001). All raw data in S1 Data. GAP, GTPase-activating protein; TBC, Tre2-Bub2-Cdc16.

TgTBC9 is one of the TBC proteins that contains a conserved dual-finger active site with the IxxDxxR and YxQ motifs (S1 Fig and Table 1). To investigate whether the active site residues are required for TgTBC9’s GAP activity, we generated complementation constructs with the full-length wild-type gene (wt), a mutant of the IxxDxxR motif (R74A), or a mutant of the YxQ motif (Q101A) (Fig 5A), which have previously been shown to disrupt function in other systems [35,57]. Complementation with the wild-type gene (TgTBC9^wt^) in TgTBC9^AID^ parasites restored the parasite’s ability to form plaques in the presence of IAA (Fig 5B and 5C). In contrast, complementation with either the TgTBC9^R74A^ or TgTBC9^Q101A^ mutant completely failed to rescue the parasite’s ability to form plaques upon knockdown of endogenous TgTBC9 (Fig 5D). Failure of the mutants to complement the knockdown was not due to expression levels, as similar levels of the TgTBC9^wt^ and the TgTBC9^R74A^ and TgTBC9^Q101A^ point mutants were confirmed by western blot analysis (Fig 5E). Quantification of these results confirmed that mutation of the TgTBC9 active sites completely disrupts the protein’s ability to rescue the lethal knockdown (Fig 5F).

### PfTBC9 partially rescues the lethal knockdown of TgTBC9 in *T*. *gondii*

**Fig 6:**
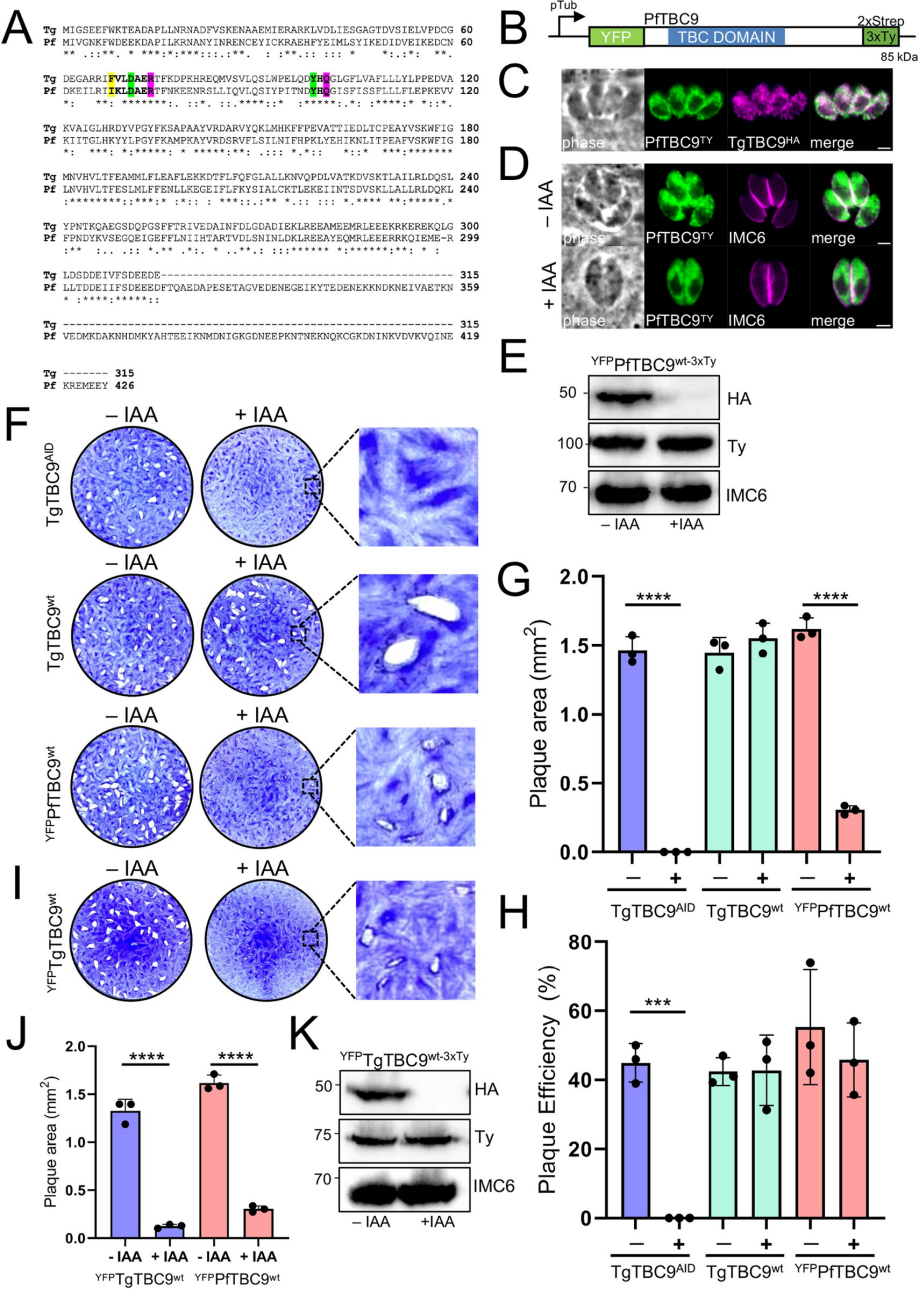
PfTBC9 partially rescues the lethal knockdown of TgTBC9. (A) Full protein alignment of TgTBC9 (Tg) and PfTBC9 (Pf) using Clustal Omega [61]. Bold residues highlighted in yellow depict semi-conserved residues; bold residues in green depict strictly conserved residues; bold residues in magenta indicate predicted R and Q catalytic sites. (B) Diagram of ^YFP^PfTBC9^wt-3xTy^ expressed from the tubulin promoter with an N-terminal YFP and C-terminal 2xStrep-3xTy tag. (C) IFA of ^YFP^PfTBC9^wt-3xTy^ colocalized with TgTBC9^AID-3xHA^ showing overlap in the ER. Green, mouse anti-Ty and GFP; magenta, rabbit anti-HA. Scale bar = 2 μm. (D) IFA of PfTBC9^wt^ with (-) or without (+) IAA for 24 h showing that PfTBC9^wt^ is expressed and unaffected in ±IAA. Magenta, mouse anti-Ty and GFP; green, rabbit anti-HA. Scale bar = 2 μm. (E) Western blot analysis of ^YFP^PfTBC9^wt-3xTy^ in the background of TgTBC9^AID^ tagged parasites. IMC6 is used as a load control. (F) Plaque assays showing that ^YFP^PfTBC9^wt^ complemented parasites formed smaller plaques compared to the TgTBC9^wt^ complemented parasites. (G) Quantification of plaque area at day 7 showing small plaque formation by ^YFP^PfTBC9^wt^ complemented parasites +IAA (****, *P <  *0.0001). (H) Quantification of plaque efficiency at day 7 show no significance between ^YFP^PfTBC9^wt^ +IAA from TgTBC9^wt^ complement groups (***, *P =* 0.0002). (I) Plaque assays showing that ^YFP^PfTBC9^wt^ complemented parasites +IAA similarly form small plaques. (J) Quantification of plaque area at day 7 showing small plaque formation by ^YFP^TgTBC9^wt^ and ^YFP^PfTBC9^wt^ complemented parasites (****, *P <* 0.0001). (K) Western blot analysis of ^YFP^TgTBC9^wt-3xTy^ in the background of TgTBC9^AID^ tagged parasites. IMC6 is used as a load control. All raw data in S1 Data. ER, endoplasmic reticulum; IFA, indirect immunofluorescence assay.

As predicted by the OrthoMCL database [33], TgTBC9 has an ortholog in *Plasmodium falciparum* (PF3D7_0904000) that we denoted PfTBC9 (S1A Fig and S1 Table) [58]. Surprisingly, the alignment between TgTBC9 and PfTBC9 reveals remarkable similarity with the exception of a 112 amino acid C-terminal extension in PfTBC9 (Fig 6A). Like its *Toxoplasma* counterpart, PfTBC9 is predicted to be essential and has the dual-finger active site [59]. To test if PfTBC9 could rescue function, we cloned a codon-optimized PfTBC9 into our complementation vector and expressed it in TgTBC9^AID^ parasites. We were unable to obtain correct expression of the protein despite using several promoters, thus we added an N-terminal YFP to the PfTBC9 construct and expressed it in the TgTBC9^AID^ strain (Fig 6B). The YFP tagged protein showed a similar ER-like pattern and was expressed at the correct size (Fig 6C–6E). In this context, PfTBC9 was only partially able to rescue the lethal phenotype resulting in the formation of smaller plaques compared to complementation with the TgTBC9^wt^ gene, but no plaque efficiency defect was observed (Fig 6F–6H). To determine if this partial rescue was a consequence of the YFP fusion, we similarly expressed TgTBC9^wt^ as a YFP fusion in the TgTBC9^AID^ strain and found that it also resulted in small plaques (Fig 6I–6K). These results indicate that the N-terminal YFP fusion partially disrupts function and that PfTBC9 is able to complement the knockdown to a similar extent as TgTBC9.

The *Trypanosoma brucei* orthologue of TgTBC9 is also a member of the RootA clade which we denote TbTBC9 (Tb427.10.7680) (S1 Fig and S1 and S2 Tables) [25]. Interestingly, TbTBC9 has a 200 amino acid N-terminal extension and also contains the dual-finger active site (S4A and S4B Fig). However, TbTBC9 is reported to localize to spotted structures at the nuclear periphery in *T*. *brucei*, which is likely the nuclear pore [60]. To determine if this protein could rescue the TgTBC9 knockdown, we also expressed it as a YFP fusion and found that it localizes to both the cytoplasm and parasite nucleus (S4C Fig). Although TbTBC9 was expressed at the correct size, plaque assays showed that the TbTBC9 protein could not rescue function of the knockdown at all (S4D–S4G Fig). This data demonstrates that while the N-terminal YFP tagged TgTBC9 and PfTBC9 can rescue function, another similar RootA orthologue is unable to do so.

### Identification of candidate Rabs that are targeted by TgTBC9

**Fig 7:**
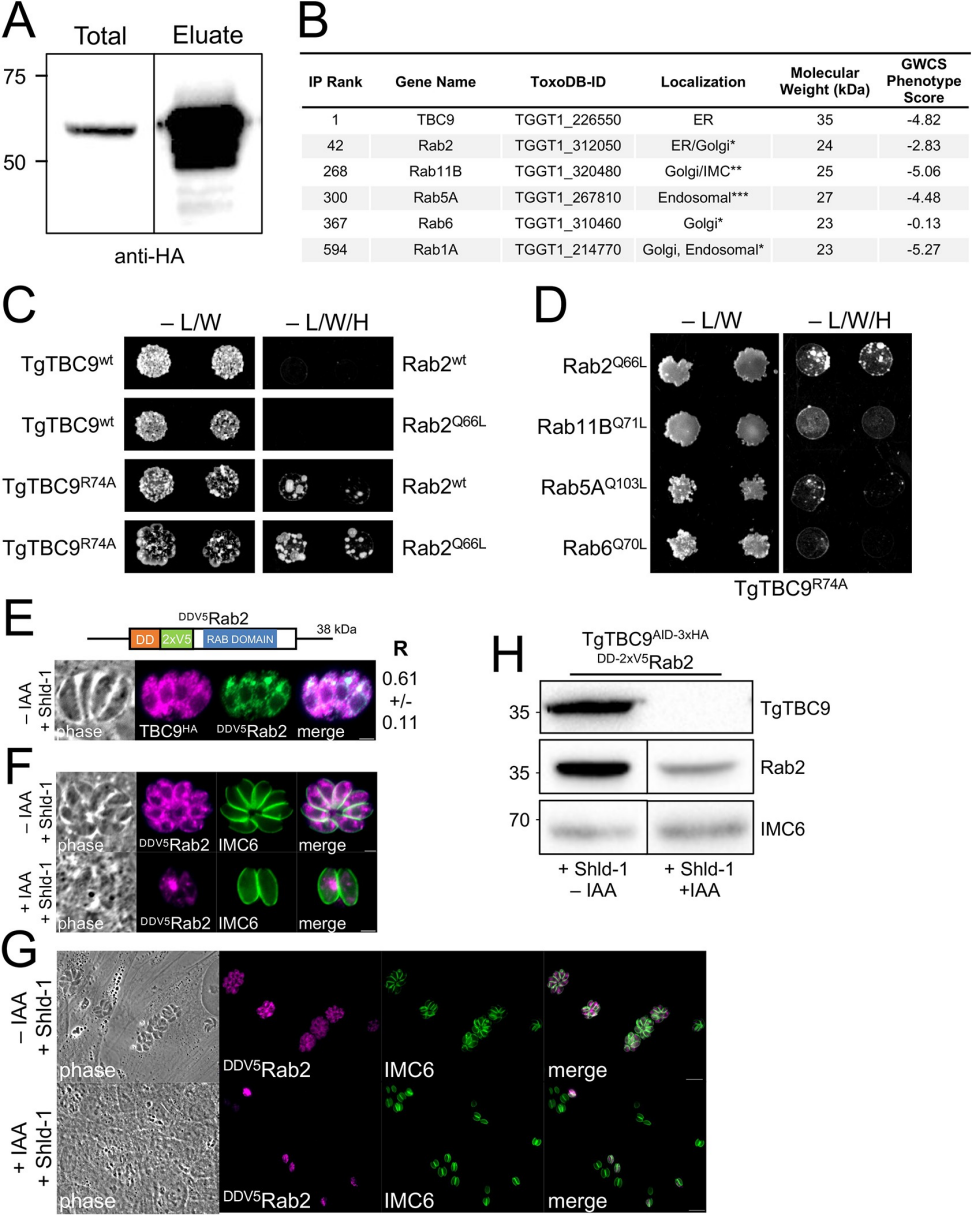
IP and pairwise Y2H of TgTBC9 reveals Rab2 an interactor. (A) Western blot analysis of the TgTBC9 IP showing the input (Total) and eluted material (Eluate) probed with mouse anti-HA antibodies. (B) Table showing the rank of TgTBC9 and small GTPase proteins from the IP analysis. The complete dataset is shown in S3 Table. Untagged parasites were used as the control. The ToxoDB-IDs, localization (* determined in [17], ** determined in [21], *** determined in [20]), molecular weight, and GWCS phenotype score are also shown [36]. (C) Spot assays of pairwise Y2H assessing TgTBC9 and Rab2 interaction using either wild-type (wt), GTP-locked mutant (Rab2^Q66L^), or catalytical inactive mutant (TgTBC9^R74A^) sequences. Yeast expressing the indicated constructs were grown under permissive (-L/W) or restrictive (-L/W/H) conditions to assess interactions. (D) Y2H assessing the interaction of catalytically inactive mutant TgTBC9 with the indicated mutant Rabs, as described in C. (E) Diagram of Rab2 showing the N-terminal DD and 2xV5 tag (^DDV5^Rab2). IFA analysis of TgTBC9^AID^ parasites expressing ^DDV5^Rab2 treated for 24 h with 1 μm Shld-1 prior to fixation. Magenta = mouse anti-HA, Green = rabbit anti-V5. Scale bars, 2 μm. Colocalizations were quantified by calculating the Pearson’s correlation coefficient (R). Mean values and standard deviation of 10–15 parasites are indicated next to the image (see also S1 Data). (F) IFA of TgTBC9^AID^ parasites expressing ^DDV5^Rab2 without (-) or with (+) IAA for 24 h (following a 4 h pretreatment ±IAA) and treated for 24 h with 1 μm Shld-1 prior to fixation showing disruption of Rab2 expression and localization. Magenta = mouse anti-V5, Green = rabbit anti-IMC6. Scale bars, 2 μm. (G) Representative IFA showing a field of TgTBC9^AID^ parasites expressing ^DDV5^Rab2 grown in ±IAA as described in F. Magenta = mouse anti-V5, Green = rabbit anti-IMC6. Scale bars, 10 μm. (H) Western blot analysis of TgTBC9^AID^ parasites expressing ^DDV5^Rab2 grown in ±IAA as descried in F showing a 52% decrease of Rab2 in IAA-treated parasites. Quantification was normalized to IMC6, which is used as a load control. GWCS, genome-wide CRIPSR screen; IFA, indirect immunofluorescence assay; IP, immunoprecipitation.

Having demonstrated that the catalytic activity of TgTBC9 is required for function, we next sought to determine which of the Rab proteins that are involved in vesicle trafficking are targeted by TgTBC9. To identify candidate Rabs as well as other potential binding partners, we carried out large-scale immunoprecipitations (IP) of TgTBC9^3xHA^ via its C-terminal epitope tag. The bound proteins were eluted using high pH and the eluates analyzed by SDS-PAGE, which showed a strong enrichment of the target protein (Fig 7A). The eluted proteins were then identified by mass spectrometry which showed that TgTBC9 was the top hit in the pull down (Fig 7B and S3 Table). We also identified Rab1A, Rab2, Rab5A, Rab6, and Rab11B as candidate small GTPase interactors. Of these candidates, Rab2 stood out as the most likely target of TgTBC9 as it was significantly more enriched in the dataset, localizes to the ER/Golgi, and has been shown to be essential for growth using an overexpression screen [17]. The mass spectrometry dataset also appeared to be enriched in secretory proteins, including a number of rhoptry, dense granule, and IMC proteins, which likely represents cargo trafficking through the secretory pathway that co-immunoprecipitated with TgTBC9 (S3 Table).

### TgTBC9 preferentially interacts with Rab2, whose localization and expression levels are affected upon TgTBC9 knockdown

To determine if TgTBC9 directly interacts with Rab2, we carried out pairwise yeast two-hybrid (Y2H) experiments [62]. The interactions of TBC proteins with their targets are often transient, thus these interactions are typically assessed using either the catalytically inactive TBC protein, a GTP-locked mutant of the Rab protein, or both mutants [35]. The rationale is that these mutants enable binding of the GAP with its partner Rab without GTP hydrolysis, leading to an improved interaction by Y2H [35]. Thus, we used both the wild-type and the catalytically inactive (R74A) mutant of TgTBC9 as bait and either the wild-type or the GTP-locked (Q66L) mutant of Rab2 as prey in the Y2H assay. Using this approach, we found that the TgTBC9^wt^ did not interact with either Rab2^wt^ or Rab2^Q66L^ (Fig 7C). However, the inactive TgTBC9^R74A^ mutant interacts with both Rab2^wt^ or Rab2^Q66L^, with the GTP-locked mutant showing stronger binding (Fig 7C). To determine if TgTBC9 interacts with other Rabs identified by the IP analysis, we created GTP locked mutants of Rabs 11B, 5A, 6, and 1A for pairwise Y2H with TgTBC9^R74A^. While Rab1A could not be assessed due to autoactivation (S5A Fig), the other Rabs showed some interaction with TgTBC9^R74A^, although the interaction did not appear as robust as with Rab2^Q66L^ (Figs 7D and S3B). This data agrees with the ranking observed by the IP analysis and suggests that while TgTBC9^R74A^ may also interact with other Rabs, it preferentially interacts with Rab2.

To directly examine the localization of TgTBC9 and Rab2, we expressed a second copy of Rab2 with an N-terminal 2xV5 tag and a destabilization domain (DD) for regulatable expression (^DDV5^Rab2). The DD targets the fusion protein for degradation unless the stabilizing compound Shield-1 (Shld1) is added to the media [63]. While overexpression of Rab2 has previously been shown to be lethal using a similar approach, expression of our construct was robust in the presence of Shld1 and did not appear to substantially affect growth [17]. We therefore used this approach to colocalize TgTBC9 and Rab2 in TgTBC9^AID-3xHA^ parasites and found colocalization in the ER as expected, although Rab2 also localizes to the Golgi as previously reported (Fig 7E) [17]. We then knocked down TgTBC9 and found that Rab2 localization was disorganized, similar to that seen with SERCA (Figs 3H and 7F). We also noticed that the overall Rab2 signal was diminished, which we confirmed by western blot analysis (52% decrease, Fig 7G and 7H). Together, this data further links TgTBC9 to Rab2 and indicates that this GAP-Rab pair collaborate to regulate ER morphology and functions in the parasite.

## Discussion

In this study, we explore the localization and function of the *T*. *gondii* TBC proteins. *Toxoplasma* contains substantially fewer TBC proteins than mammalian cells, which agrees with apicomplexans only containing a reduced set of core Rab proteins [18,64]. This smaller number of regulators and their targets may simplify characterization of how these proteins regulate protein trafficking throughout the secretory pathway and to the unique organelles of the parasite. Of the 18 predicted TBC proteins in *T*. *gondii*, 16 are expressed in tachyzoites with various localizations, including the ER, Golgi, cytoplasmic vesicles, and maternal and daughter bud IMC. Interestingly, the localizations of the TBC proteins in *Toxoplasma* align well with that of the Rabs, which have also been shown to localize to the ER, Golgi, cytoplasmic vesicles, and IMC [17,20–22]. It is also intriguing that the TBC and Rab proteins are not present in the downstream secretory organelles (i.e., micronemes, rhoptries, or dense granules), suggesting other regulators mediate vesicular traffic in these compartments. Ultimately, determining the precise function of each TBC protein and identifying their target(s) will be important for a complete understanding of secretory and vesicular traffic in *T*. *gondii* and related parasites.

The fact that the number of TBC proteins exceeds that of the Rabs and that many of the TBC proteins are predicted to be dispensable suggests that there are redundancies among family members [17,36]. Redundancy would be most likely in regions of the parasite that contain multiple family members such as the Golgi, cytoplasmic vesicles, or daughter IMC, indicating that multiple knockouts may be required to assess function. One likely candidate for redundancy is the TBS proteins TgTBC16 and TgTBC17, which both localize to cytoplasmic vesicles and are each predicted to be dispensable [32,36]. Another possibility is TgTBC11 and TgTBC15, which localize to IMC daughter buds indicating a role in replication; however, TgTBC11 has a −2.83 phenotype score in the GWCS, suggesting disruption of its gene alone may significantly impair the parasites [36]. Other proteins with relatively negative phenotype scores include TgTBC18 (−2.86), TgTBC13 (−2.04), and TgTBC2 (−1.84) which may yield significant phenotypes when disrupted individually; however, they also have other family members nearby that may compensate or partially compensate for their absence [36]. We also cannot exclude the possibility that other regulators lacking TBC domains exist, some TBC proteins carry out other functions, or the intrinsic GTPase activities of some of the Rabs are sufficient for function [16,27,65].

While many TBC proteins in both *T*. *gondii* and other systems appear to be dispensable [25,26,45], we demonstrate here that TgTBC9 is essential for growth in vitro. TgTBC9 is the smallest member of the family and largely consists of its TBC domain, which we show requires both of its conserved active site residues for function. Conditional knockdown of the protein arrests parasite growth, disrupts the organization of the ER and mitochondria, and results in the formation of large LDs and ER encapsulated structures, but it does not have a gross effect on downstream secretory compartments such as the Golgi, micronemes, and rhoptries. The formation of large LDs and other ER-related structures is intriguing as lipid droplet synthesis occurs in the ER and these structures probably occur from the aberrant activity of the TgTBC9-Rab2 [51]. This misregulation of lipid homeostasis then likely results in the activation of lipolysis or autophagy pathways seeking to minimize lipotoxicity [66,67]. The growth arrest we observe may be a consequence of a disruption of the earliest stages of secretory traffic that results in an ER stress feedback loop [68], which blocks replication as well as the delivery of newly synthesized cargo from entering into the secretory pathway. Our identification of at least 2 other ER family members indicates that these TBC proteins cannot compensate for the loss of TgTBC9 and likely carry out distinct functions in the ER.

Phylogenetic analyses of TBC proteins have proven challenging, likely because of ancient divergences among the ancestral clades present in the most recent common ancestor of eukaryotes, which results in homoplasy and problems with alignment hampering phylogenetic inference [25]. Ancestral TBC clades have experienced differential retention and diversification in different eukaryotic lineages, and as a result the phylogenetic relations of homologs within particular TBC clades is less difficult to infer, typically following the phylogenetic relationships of the taxa of origin. Our phylogeny of TBC-RootA proteins confirms that TgTBC9 belongs to the “RootA” clade of TBC proteins, which was first described in studies examining *T*. *brucei* TBC proteins and appears to be restricted to protozoans [25]. The *Plasmodium* orthologue of TgTBC9 is well conserved, predicted to be essential, and can rescue the lethal TgTBC9 knockdown, suggesting this protein has a conserved function in apicomplexans [59]. This is likely not the case for the *T*. *brucei* family member which has been experimentally determined to be nuclear in *T*. *brucei* and cannot rescue the TgTBC9 knockdown [60]. It will be interesting to determine the localizations and functions of the other RootA family members to determine how this clade has diversified to carry out distinct functions in the protozoa.

We used IP and Y2H analyses to identify Rab2 as a preferred binding partner and likely target of TgTBC9. Rab2 is a good fit for a TgTBC9 target as it has been shown to localize to the early secretory pathway (ER, Golgi) and is known to control anterograde traffic between the ER and Golgi [17,69]. In addition, overexpression of Rab2 in *T*. *gondii* indicates that it is essential for growth with no apparent effects the micronemes or rhoptries [17]. Rab2 has also been shown to be involved in the regulation of lipophagy and for promoting autophagy [66], further indicating that the TgTBC9-Rab2 pair is important for maintaining ER functions and the phenotypes we observe upon TgTBC9 knockdown. Since some TBC proteins in other systems are able to act on multiple Rabs, it is also possible TgTBC9 also regulates some of the other Rabs in our IP and Y2H experiments [67,70]. Rab5A, Rab6, and Rab11B all showed some level of interaction in the Y2H assay [17,20,21]. However, these proteins localize to Golgi or endosomal compartments in *Toxoplasma*, and there have been reports that some TBC-Rab pairs interact promiscuously in Y2H assays [20,21,67,71]. Ultimately, understanding the precise pairing of Rabs with their TBC regulators is likely best determined using multiple assays including localization, IP, Y2H, and in vitro GTPase activity assays [67,72].

Taken together, this study sheds light on the localization and function of TBC proteins in *T*. *gondii*. Characterization of the TBC proteins contributes to a broader understanding of the secretary pathway and how proteins are trafficked in the parasite. While redundancy may exist in certain regions of the secretory or vesicular trafficking pathways, we demonstrate that TgTBC9 is essential for growth in vitro and plays a crucial role in regulating the early stages of secretory traffic and other ER functions such as LD biogenesis and lipophagy. Our findings also indicate that TgTBC9 directly targets Rab2, a key GTPase involved in ER-Golgi trafficking and lipid homeostasis. Because TgTBC9 is protozoan-specific and may have activities that are distinct from its mammalian counterparts, it could serve as a new therapeutic target that can be used to combat *T*. *gondii* and related apicomplexan parasites.

### Inclusion and diversity

We support inclusive, diverse, and equitable conduct of research.

## Materials and methods

### *Toxoplasma* and host cell culture

Parental *T*. *gondii* RHΔ*ku80*Δ*hpt* and modified strains were grown on confluent monolayers of human foreskin fibroblasts (HFFs) host cells at 37°C and 5% CO_2_ in Dulbecco’s modified Eagle’s medium (DMEM) supplemented with 5% fetal bovine serum (Gibco), 5% Cosmic Calf serum (HyClone), and penicillin-streptomycin-l-glutamine (Gibco). Constructs containing selectable markers were selected using 40 μm chloramphenicol (CAT), 1 μm pyrimethamine, or 50 μg/ml mycophenolic acid-xanthine (HXGPRT) [73–75]. Homologous recombination to the UPRT locus was negatively selected using 5 μm 5-fluorodeoxyuridine (FUDR) [76].

### Sequence analysis and phylogenetic inference

Sequences for TgTBC1-18 from *T*. *gondii* were retrieved from ToxoDBv43 (https://toxodb.org/toxo/) and were assessed for TBC and other domains by NCBI conserved domain search tool, SMART, and Interpro [28–30]. Ortholog groups were retrieved from OrthoMCL database (https://orthomcl.org/orthomcl/app) and further assessed by NCBI BLAST analysis [33,34]. Accession numbers for RootA sequences are given in S2 Table and multiple sequence alignments were generated in Geneious Prime 2021.2.2. (https://www.geneious.com). Maximum likelihood phylogenetic inference of the approximately 200 amino acid TBC domain of the RootA TBC proteins was preformed in RAxML using the LG+G4 amino acid substitution model, estimating support over 1,000 bootstrap replicates, and results were visualized by the Interactive Tree of Life (iTOL) program [77,78]. Protein alignments were generated using Clustal Omega [61].

### Chemicals and antibodies

3-Indoleacetic acid (IAA; Sigma-Aldrich; I2886) was used at 500 μm from a 500 mM stock in 100% ethanol. The ligand Shield-1 (Shld-1) was used at 1 μm from a 1 mM stock in 100% ethanol [63]. Neutral lipids were stained using 40 μm of BODIPY 493/503 (#D3922; Invitrogen) from a 40 mM stock in DMSO, as previously described [50]. The hemagglutinin (HA) epitope was detected with mouse monoclonal antibody (mAb) anti-HA (HA.11) (BioLegend) or rabbit mAb anti-HA (C29F4, Cell Signaling). The Ty1 epitope was detected with mouse mAb BB2 [79], V5 epitope was detected with mouse mAb anti-V5 (#R96025; Invitrogen), and the OLLAS tag was detected using the rat mAb anti-OLLAS [80]. The c-Myc epitope was detected with mouse anti-Myc (mAb 9E10) or rabbit polyclonal antibody (pAb) (#PA1981; Invitrogen). *Toxoplasma*-specific antibodies include rabbit pAb anti-IMC6 [81], mouse anti-SERCA [40], mouse mAb anti-F1β subunit (5F4) [82], mouse mAb anti-ATrx1 (11G8) [83], mouse mAb anti-MIC2 [84], mouse mAb anti-ROP7 (1B10) [85], mouse pAb anti-Gra14 [85], mouse anti-IMC1 [86], and guinea pig anti-NHE3 [12].

### Immunofluorescence assays and western blotting

HFF cells were grown to confluency on glass coverslips and infected with *T*. *gondii* parasites expressing previously mentioned epitope tags. After time periods ranging between 18 and 36 h, infected coverslips were fixed using 3.7% formaldehyde and processed for indirect immunofluorescence assay (IFA) as described previously [87]. Primary antibodies were detected by species-specific secondary antibodies conjugated to Alexa594/488. Coverslips were mounted in Vectashield (Vector Labs) and viewed with an Axio Imager.Z1 fluorescence microscope (Zeiss). Images were processed with the ZEN 3.7 software (Zeiss), which included deconvolution and adaptation of the magenta pseudocolor from the 594 fluorophores. The Pearson’s correlation coefficient was calculated using ZEN 3.7 software (Zeiss) [41].

For western blotting, parasites were lysed in 1× Laemmli sample buffer (50 mM Tris-HCl [pH 6.8], 10% glycerol, 2% SDS, 100 mM DTT, 0.1% bromophenol blue) and boiled at 100°C for 10 min. Lysates were resolved by SDS-PAGE and transferred to nitrocellulose membranes. Protein blots were probed with the appropriate primary antibody followed by the corresponding secondary antibodies conjugated to horse radish peroxidase (HRP). Proteins were visualized by chemiluminescence (Thermo Scientific) and imaged on ChemiDoc XRS+ (Bio-Rad).

### Epitope tagging

For endogenous C-terminal tagging, guides approximately 100 to 200 bp downstream of each gene’s stop codon were ligated into a pU6-Universal plasmid, as described previously [88]. A homology-directed repair (HDR) template was amplified from LIC (ligation-independent cloning) epitope-tagging vectors (e.g., 3xHA, smHA, mIAA7-3xHA, 3xMyc, smMyc, 3xV5, 3xTy, and smOLLAS) with 40-bp flanking regions for recombination at the 3′ untranslated region (UTR) of each gene and a selection cassette [89]. This template was PCR amplified in a 400 μl total reaction, purified by phenol-chloroform extraction, precipitated using ethanol, and electroporated into RHΔ*hxgprt*Δ*ku80* parasites, along with 50 μg of the sequence-verified pU6-Universal plasmid. Confluent monolayers of HFF cells were infected with transfected parasites, and appropriate drug selection (containing either 1 μm pyrimethamine, 50 μg/ml mycophenolic acid/xanthine, or 1 μm chloramphenicol) was applied. Parasites that underwent successful tagging were screened by IFA, and clonal lines of tagged parasites were obtained through limiting dilution. TgTBC1-18 were tagged using CRISPR/Cas9 with primers P1 to P72 and verified by PCR using primers P73 to P92 (S4 Table). TgGLP2, TgVps9, Der1-2, TgCep250, and IMC29 were tagged with primers P147 to P166 (S4 Table) [23,42,47,90,91].

### Plaque assays

For plaque assays, six-well plates with HFF monolayers were allowed to reach confluency. Equivalent number of parasites were grown on confluent HFF monolayers ± 500 μm IAA. Parasite plaques were allowed to form for 7 days, after which cells were fixed with ice-cold methanol and stained with crystal violet [92]. The areas of 50 plaques per condition was measured using ZEN software (Zeiss). All plaque assays were performed in triplicate using biological replicates. Statistical significance was calculated using a two-sample two-tailed *t* test. Graphs and figures were generated using Prism GraphPad 8.0.

### Complementation and mutant construct generation

To generate the TgTBC9 wild-type complement construct, the entire coding region of the gene was PCR amplified from cDNA and cloned into a UPRT-locus knockout vector driven by the TgGT1_238895 promoter, as previously described (primers P93-P96; S4 Table) [62]. The online NEBasechanger (https://nebasechanger.neb.com) was used to design the primers. Mutant constructs were built with the complementation vector as a template using the Q5 Site-Directed Mutagenesis Kit (NEB) to mutate specific amino acids (primers P97-P100; S4 Table). The plasmids constructs were linearized with PsiI-v2 (NEB), transfected into the TgTBC9^AID^ parasites along with a universal pU6 tagging to the UPRT coding region, and selected with 5 μg/ml 5-Fluoro-5′-deoxyuridine (FUDR), as previously described [76]. TgTBC9^wt-3xTy^, TgTBC9^R74A-3xTy^, or TgTBC9^Q101A-3xTy^ expressing clones were confirmed by IFA.

The TbTBC9 complementation construct was amplified from genomic DNA and cloned into a UPRT-locus knockout vector driven from a tubulin promoter (primers P109-P112; S4 Table). The PfTBC9 gene was codon optimized using the Codon Optimization Tool (https://www.idtdna.com/pages/tools/codon-optimization-tool) and synthesized by IDT. The syntenic gene block was cloned into UPRT-locus vectors (primers P101-P108; S4 Table). To generate the ^DDV5^Rab2 construct, full-length cDNA of Rab2 was cloned into a UPRT-locus knockout vector with an N-terminal DD-2xV5 tag [63] (primers P167-P170; S4 Table).

### Transmission electron microscopy

For ultrastructural observations, *T*. *gondii*-infected HFF grown as monolayers on a 6-well dish were exposed to 500 μm IAA or ethanol solvent before fixation 24 h postinfection in 2.5% glutaraldehyde in 0.1 mM sodium cacodylate (pH 7.4) and processed as described previously [93]. Ultrathin sections of infected cells were stained with osmium tetraoxide before examination with Hitachi 7600 EM under 80 kV equipped with a dual AMT CCD camera system.

### Immunoprecipitation

For immunoprecipitation, TgTBC9^3xHA^ or control parasites (RHΔ*hpt*Δ*ku80*) were used to infect 10 T150 plates with confluent monolayers of HFF. Extracellular parasites were collected, washed in PBS, and lysed using NP-40 lysis buffer (50 mM Tris [pH 8.0], 150 mM NaCl, 1% NP-40) supplemented with Complete Protease Inhibitor Cocktail (Roche) for 30 min on ice. Lysates were centrifuged at 10,000 x *g* for 15 min to pellet and remove insoluble material after which the remaining soluble lysate was incubated with anti-HA sepharose beads (Roche) for 4 h at room temperature. The beads were collected by centrifugation and washed 4 times using NP-40 lysis buffer. Eluted proteins were analyzed by western blot, and the remaining material was analyzed by mass spectrometry.

### Sample preparation and LC-MS acquisition and analysis

The protein pellets were resuspended with 100 μl digestion buffer (8 M Urea, 0.1 M Tris-HCl [pH 8.5]), incubated in RT for 30 min. Each sample was reduced and alkylated via sequential 20-min incubations with 5 mM TCEP and 10 mM iodoacetamide at room temperature in the dark while being mixed at 1,200 rpm in an Eppendorf thermomixer, and 10 μl of carboxylate-modified magnetic beads (CMMBs and also widely known as SP3) was added to each sample [94]. Ethanol was added to a concentration of 50% to induce protein binding to CMMB. CMMB were washed 3 times with 80% ethanol and then resuspended with 50 μl 50 mM TEAB.

The protein was digested overnight with 0.1 μg LysC (Promega) and 0.8 μg trypsin (Thermo Scientific, 90057) at 37°C. Following digestion, 1.2 ml of 100% acetonitrile was added to each to sample to increase the final acetonitrile concentration to over 95% to induce peptide binding to CMMB. CMMB were then washed 3 times with 100% acetonitrile and the peptide was eluted with 65 μl of 2% DMSO. Eluted peptide samples were dried by vacuum centrifugation and reconstituted in 5% formic acid before analysis by LC-MS/MS.

Peptide samples were separated on a 75 μm ID, 25 cm C18 column packed with 1.9 μm C18 particles (Dr. Maisch GmbH) using a 140-min gradient of increasing acetonitrile concentration and injected into a Thermo Orbitrap-Fusion Lumos Tribrid mass spectrometer. MS/MS spectra were acquired using data-dependent acquisition (DDA) mode. MS/MS database searching was performed using MaxQuant (1.6.10.43) against the *T*. *gondii* GT1 reference proteome from ToxoDB [95].

### Pairwise yeast two-hybrid

Wild-type or the GTP-fixed mutant of Rab2, 11B, 5A, 6, and 1A lacking their C-terminal prenylation sites were cloned into the pP6 vector (Hybrigenics SA) as N-terminal fusions with the GAL4 activating domain using Gibson assembly (primers P117-P136; S4 Table). GTP-locked mutant TgTBC9^R74A^ or wild-type TgTBC9 were cloned into the pB27 vector (Hybrigenics SA) as N-terminal fusions with the LexA DNA-binding domain (primers P113-P116; S4 Table). The Rab2, 11B, 5A, 6, and 1A mutant constructs were produced by site-directed mutagenesis as described above (primers P137 and P146; S4 Table). The following Rab mutants were used in this study: Rab1A (Q67L), Rab2(Q66L), Rab5A(Q103L), Rab6(Q70L), and Rab11B(Q71L). To test for interactions, pairs of constructs were transformed into the L40 strain of *S*. *cerevisiae* [MATa his3Δ200trp1-901 leu2-3112 ade2 LYS2::(4lexAop-HIS3) URA3::(8lexAop-lacZ) GAL4]. Strains were grown overnight in permissive (−Leu/−Trp) medium, diluted to an OD_600_ of 2, and spotted in 2× dilutions in both permissive and restrictive (−Leu/−Trp/−His) media. Relative growth in the 2 conditions was assessed after 6 days incubation at 30°C.

## Supporting information

S1 Fig TgTBC1-18 contain orthologs in other systems and most retain the TBC dual-finger active site. (A) Diagram of TgTBC1-18 showing orthologs in other species indicated. Blue checks indicate orthologs determined by OrthoMCL, while orange checks indicate likely orthologs based on NCBI BLAST analysis. (B) Clustal Omega alignment with the TBC domain region of TgTBC1, 3, 4, 7, 8, 9, 10, 14, 15, and 18 sequences showing TBC dual-finger active site IxxDxxR and YxQ. Bold residues highlighted in yellow depict semi-conserved residues; bold residues in green depict strictly conserved residues. Red triangles indicate the conserved R and Q resides important for catalytic activity. Asterisks (*) indicate identity, colon (:) indicates highly conserved residues, and a period (.) indicates weakly conserved residues.
(TIF)

S2 Fig Organelles unaffected by knockdown of TgTBC9. (A) IFA of TgTBC9^AID^ without (-) or with (+) IAA for 24 h (following a 4 h pretreatment in ±IAA) showing that the micronemes are unaffected using anti-MIC2. Magenta, mouse anti-MIC2; green, rabbit anti-IMC6. (B) IFA of TgTBC9^AID^ parasites grown as described in A but staining for rhoptries using anti-ROP7. Magenta, mouse anti-ROP7; green, rabbit anti-IMC6. (C) TgTBC9^AID^ parasites grown as described in A but with staining for dense granules using anti-GRA14. Magenta, mouse anti-GRA14; green, rabbit anti-IMC6. (D) TgTBC9^AID^ parasites grown as described in A but with staining for apicoplast using anti-Atrx1. Magenta, mouse anti-Atrx1; green, rabbit anti-IMC6. (E) TgTBC9^AID^ parasites grown as described in A but with staining for centrosomes using an endogenously tagged Cep250^3xV5^ strain. Magenta, mouse anti-V5; green, rabbit anti-IMC6. (F) TgTBC9^AID^ parasites grown as described in A but with staining for the ELC using an endogenously tagged Vps9^3xV5^ strain. Magenta, mouse anti-V5; green, rabbit anti-IMC6. (G) TgTBC9^AID^ parasites grown as described in A but with staining for the IMC. Magenta, mouse anti-IMC1; green, rabbit anti-IMC6. (H) TgTBC9^AID^ parasites grown as described in A but with staining for the PLVAC using anti-NHE3. Magenta, guinea pig anti-NHE3; green, rabbit anti-IMC6. (I) TgTBC9^AID^ parasites grown as described in A but with staining for Golgi using an endogenously tagged GLP2^3xV5^ strain. Magenta, mouse anti-V5; green, rabbit anti-IMC6. (J) TgTBC9^AID^ parasites grown as described in A but with staining for the DrpB using anti-DrpB. Magenta, rat anti-DrpB; green, rabbit anti-IMC6. Scale bars for all images, 2 μm. (K) Western blot analysis of showing ROP7 protein levels are unaffected upon IAA treatment. IMC6 is used as a load control. (L) Western blot analysis of showing MIC2 proteins levels upon IAA treatment. IMC6 is used as a load control.
(TIF)

S3 Fig Ultrastructure of TgTBC9 knockdown parasites. (A–C) TEM of intracellular *Toxoplasma* in HFF for 24 h in the presence of IAA. (A) Representative TEM images of vacuoles containing 2 parasites, highlighting different morphologies based on parasite body shape, ranging from normal to dramatically aberrant (panels a to c). The quantification of normal shape, slightly misshaped, and severely misshaped parasites observed from 86 sections of vacuoles is shown. (B) In panel a: TEM showing canonical lipid droplets (LD) surrounded by ER tubules (solid frame) and abnormal membranous assemblies (dotted frame) in the cytoplasm of the same parasite. Panels b and c show examples of LD surrounded by ER tubules and abnormal membranous assemblies at higher magnification. (C) TEM showing examples of cytoplasmic clefts (arrowheads), often initiated at the nuclear envelope as seen in panel a and more pronounced in panel b. n, nucleus; PV, parasite vacuoles.
(TIF)

S4 Fig TbTBC9 does not rescue the lethal knockdown of TgTBC9. (A) Diagram showing alignment of TbTBC9, TgTBC9, and PfTBC9 revealing N-terminal extension in TbTBC9 and C-terminal extension in PfTBC9. (B) Full protein alignment of TbTBC9 (Tb), TgTBC9 (Tg), and PfTBC9 (Pf) using Clustal Omega [61]. Bold residues highlighted in yellow depict semi-conserved residues; bold residues in green depict strictly conserved residues; bold residues in magenta indicate R and Q residues important for catalytic activity. (C) IFA of ^YFP^TbTBC9^wt-3xTy^ colocalized with TgTBC9^AID-3xHA^ showing overlap. Green, mouse anti-Ty; magenta, rabbit anti-HA. Scale bar = 2 μm. D) IFA of TbTBC9^wt^ with (-) or without (+) IAA for 24 h showing that TbTBC9^wt^ is expressed in the presence and absence of IAA. Magenta, mouse anti-Ty; green, rabbit anti-HA. Scale bar = 2 μm. (E) Western blot analysis of ^YFP^TbTBC9^wt-3xTy^ in the background of TgTBC9^AID^ tagged parasites. IMC6 is a loading control. (F) Plaque assays showing that TbTBC9^wt^ complemented parasites +IAA fail to form plaques. (G) Quantification of plaque area at day 7 showing no plaque formation by TbTBC9^wt^ complemented parasites +IAA (****, *P <* 0.0001). All raw data in S1 Data.
(TIF)

S5 Fig Control Y2H experiments. (A) Y2H of Rab1A^Q67L^ with corresponding empty vector showing autoactivation. (B) Spot assays of pairwise Y2H demonstrates a lack of autoactivation of the indicated constructs. Each construct is coexpressed with the corresponding empty bait or prey vectors, as appropriate.
(TIF)

S1 Table Overview of T. gondii TBC proteins and their orthologs. Designation, gene IDs, and ortholog groups of TBC1-18.
(XLSX)

S2 Table TBC-RootA Sequences. Accession numbers, full protein sequences, TBC domain range, TBC domain sequences, and protein length for all TBC-RootA proteins.
(XLSX)

S3 Table Co-immunoprecipitation and mass spectrometry results. (XLSX)

S4 Table Primers used in this study. (XLSX)

S1 Data All numerical input data for main and supporting figures. (XLSX)

S1 Raw Images Original images for blots and gels. (PDF)

## Abbreviations

AID: auxin-inducible degron
CMMB: carboxylate-modified magnetic bead
DD: destabilization domain
DDA: data-dependent acquisition
DMEM: Dulbecco’s modified Eagle’s medium
ELC: endosome-like compartment
ER: endoplasmic reticulum
GAP: GTPase-activating protein
GEF: guanine nucleotide exchange factor
GWCS: genome-wide CRIPSR screen
HDR: homology-directed repair
HFF: human foreskin fibroblast
HRP: horse radish peroxidase
IFA: indirect immunofluorescence assay
IMC: inner membrane complex
IP: immunoprecipitation
iTOL: Interactive Tree of Life
LD: lipid droplet
LIC: ligation-independent cloning
PLVAC: plant-like vacuolar compartment
PV: parasitophorous vacuole
smHA: spaghetti monster HA
smOLLAS: spaghetti monster OLLAS
TBC: Tre2-Bub2-Cdc16
TEM: transmission electron microscopy
UTR: untranslated region
