# Supplementary figures and images for: Systematic characterization of all Toxoplasma gondii TBC domain-containing proteins identifies an essential regulator of Rab2 in the secretory pathway
Source: PLoS Biol. 2024 May 7;22(5):e3002634. doi: 10.1371/journal.pbio.3002634 (PMC11101121; doi:10.1371/journal.pbio.3002634)

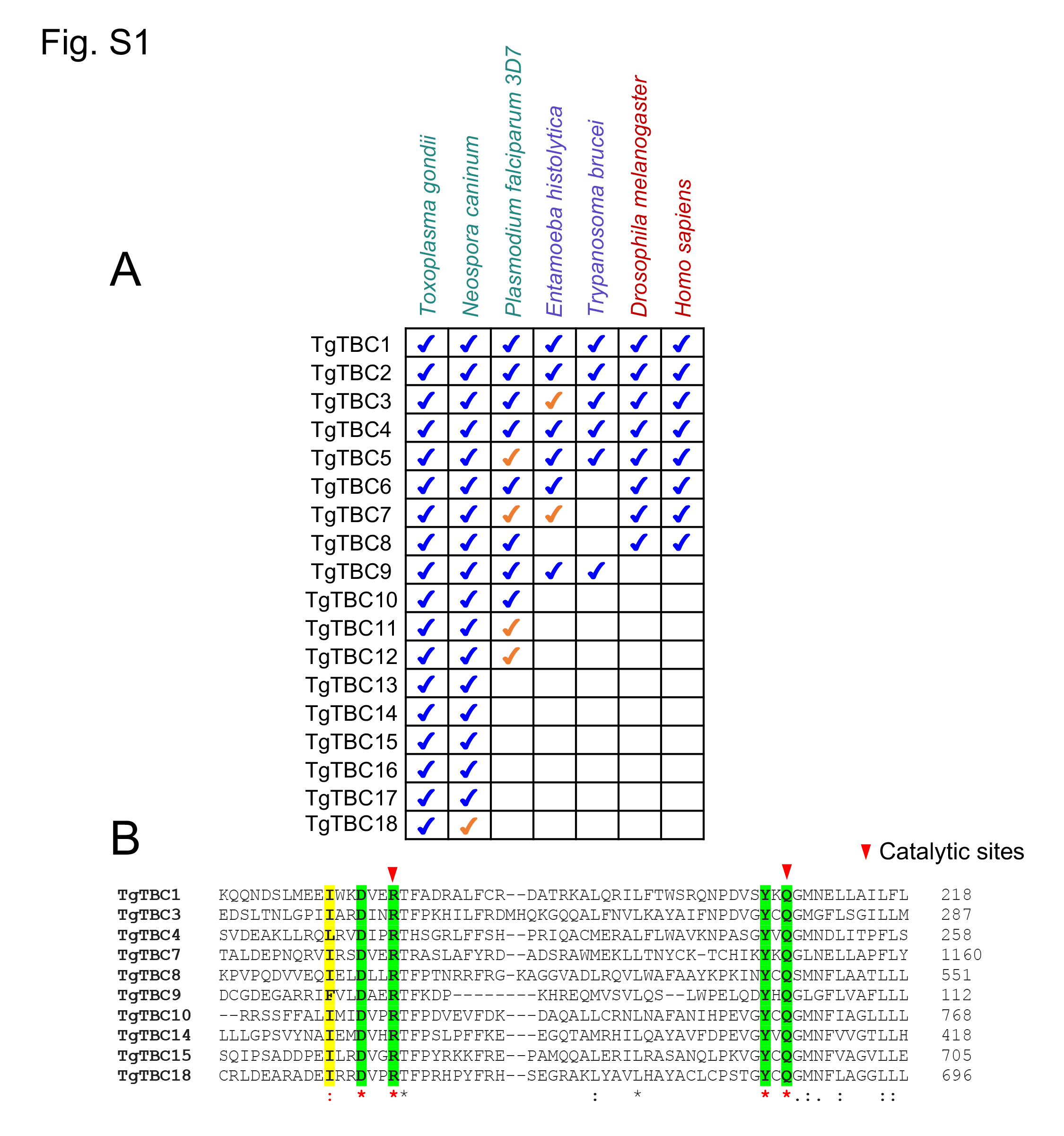

Supplement: S1 Fig — (A) Diagram of TgTBC1-18 showing orthologs in other species indicated. Blue checks indicate orthologs determined by OrthoMCL, while orange checks indicate likely orthologs based on NCBI BLAST analysis. (B) Clustal Omega alignment with the TBC domain region of TgTBC1, 3, 4, 7, 8, 9, 10, 14, 15, and 18 sequences showing TBC dual-finger active site IxxDxxR and YxQ. Bold residues highlighted in yellow depict semi-conserved residues; bold residues in green depict strictly conserved residues. Red triangles indicate the conserved R and Q resides important for catalytic activity. Asterisks (*) indicate identity, colon (:) indicates highly conserved residues, and a period (.) indicates weakly conserved residues. (TIF) [file pbio.3002634.s001.tif]

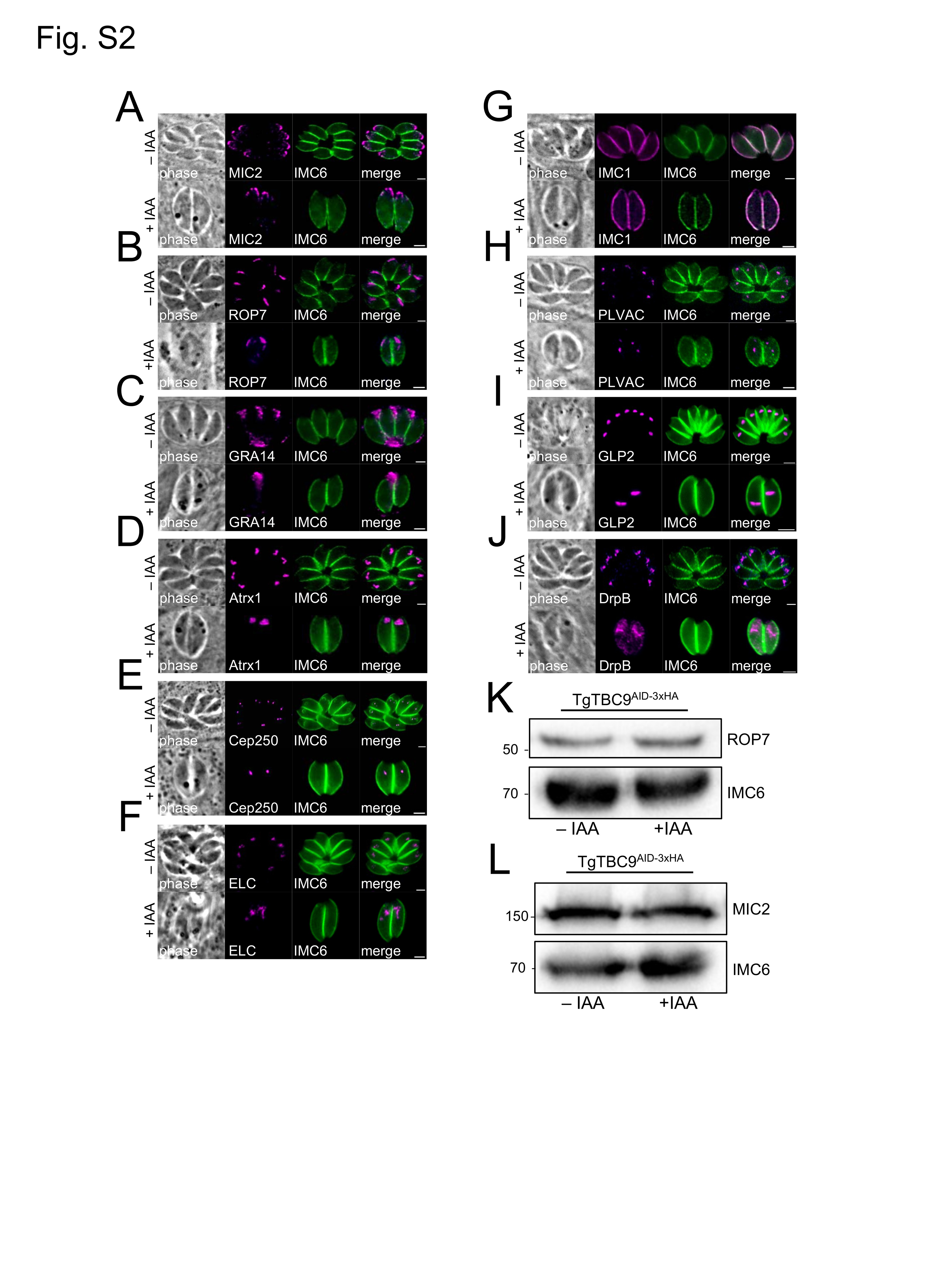

Supplement: S2 Fig — (A) IFA of TgTBC9AID without (-) or with (+) IAA for 24 h (following a 4 h pretreatment in ±IAA) showing that the micronemes are unaffected using anti-MIC2. Magenta, mouse anti-MIC2; green, rabbit anti-IMC6. (B) IFA of TgTBC9AID parasites grown as described in A but staining for rhoptries using anti-ROP7. Magenta, mouse anti-ROP7; green, rabbit anti-IMC6. (C) TgTBC9AID parasites grown as described in A but with staining for dense granules using anti-GRA14. Magenta, mouse anti-GRA14; green, rabbit anti-IMC6. (D) TgTBC9AID parasites grown as described in A but with staining for apicoplast using anti-Atrx1. Magenta, mouse anti-Atrx1; green, rabbit anti-IMC6. (E) TgTBC9AID parasites grown as described in A but with staining for centrosomes using an endogenously tagged Cep2503xV5 strain. Magenta, mouse anti-V5; green, rabbit anti-IMC6. (F) TgTBC9AID parasites grown as described in A but with staining for the ELC using an endogenously tagged Vps93xV5 strain. Magenta, mouse anti-V5; green, rabbit anti-IMC6. (G) TgTBC9AID parasites grown as described in A but with staining for the IMC. Magenta, mouse anti-IMC1; green, rabbit anti-IMC6. (H) TgTBC9AID parasites grown as described in A but with staining for the PLVAC using anti-NHE3. Magenta, guinea pig anti-NHE3; green, rabbit anti-IMC6. (I) TgTBC9AID parasites grown as described in A but with staining for Golgi using an endogenously tagged GLP23xV5 strain. Magenta, mouse anti-V5; green, rabbit anti-IMC6. (J) TgTBC9AID parasites grown as described in A but with staining for the DrpB using anti-DrpB. Magenta, rat anti-DrpB; green, rabbit anti-IMC6. Scale bars for all images, 2 μm. (K) Western blot analysis of showing ROP7 protein levels are unaffected upon IAA treatment. IMC6 is used as a load control. (L) Western blot analysis of showing MIC2 proteins levels upon IAA treatment. IMC6 is used as a load control. (TIF) [file pbio.3002634.s002.tif]

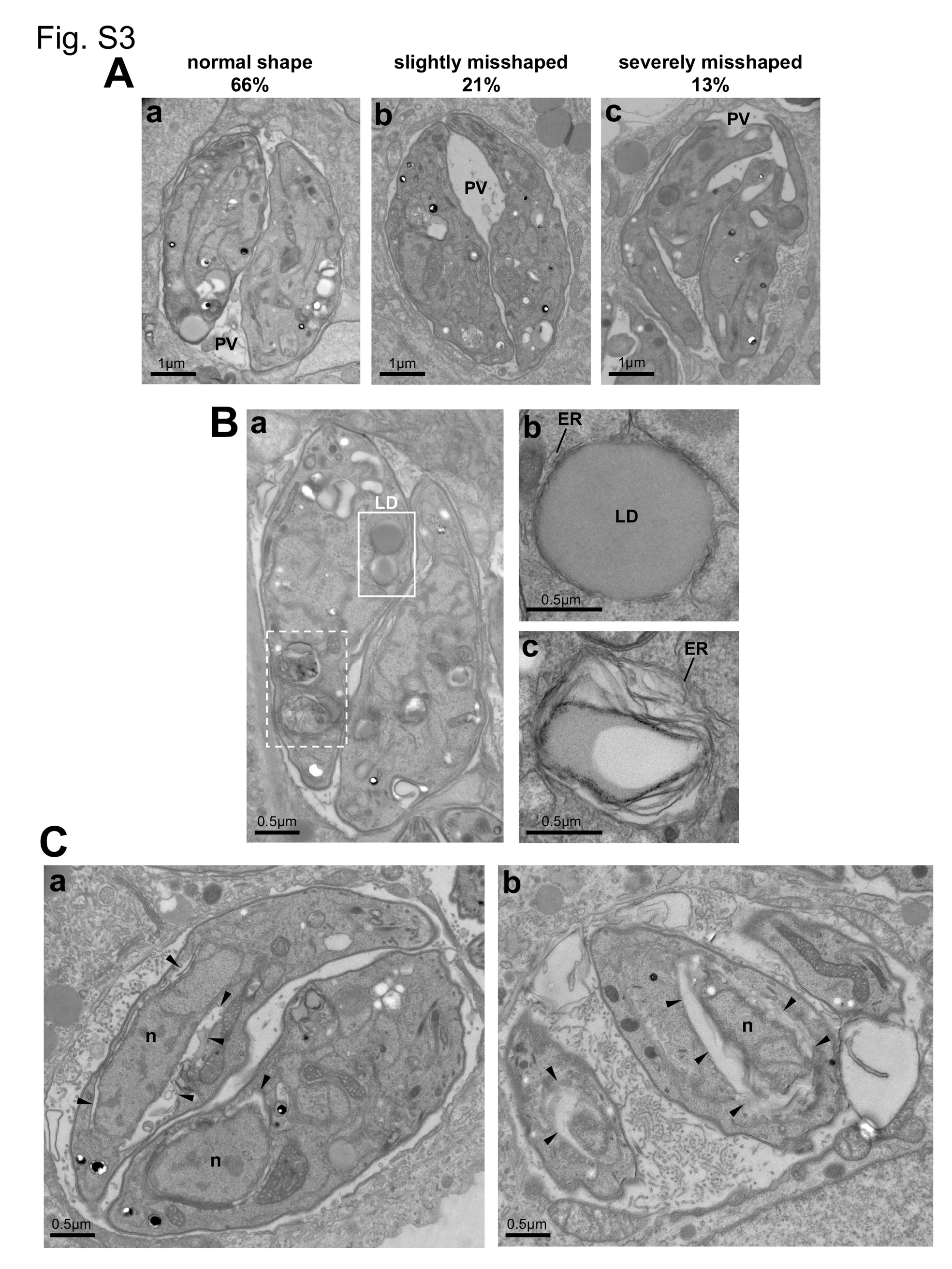

Supplement: S3 Fig — (A–C) TEM of intracellular Toxoplasma in HFF for 24 h in the presence of IAA. (A) Representative TEM images of vacuoles containing 2 parasites, highlighting different morphologies based on parasite body shape, ranging from normal to dramatically aberrant (panels a to c). The quantification of normal shape, slightly misshaped, and severely misshaped parasites observed from 86 sections of vacuoles is shown. (B) In panel a: TEM showing canonical lipid droplets (LD) surrounded by ER tubules (solid frame) and abnormal membranous assemblies (dotted frame) in the cytoplasm of the same parasite. Panels b and c show examples of LD surrounded by ER tubules and abnormal membranous assemblies at higher magnification. (C) TEM showing examples of cytoplasmic clefts (arrowheads), often initiated at the nuclear envelope as seen in panel a and more pronounced in panel b. n, nucleus; PV, parasite vacuoles. (TIF) [file pbio.3002634.s003.tif]

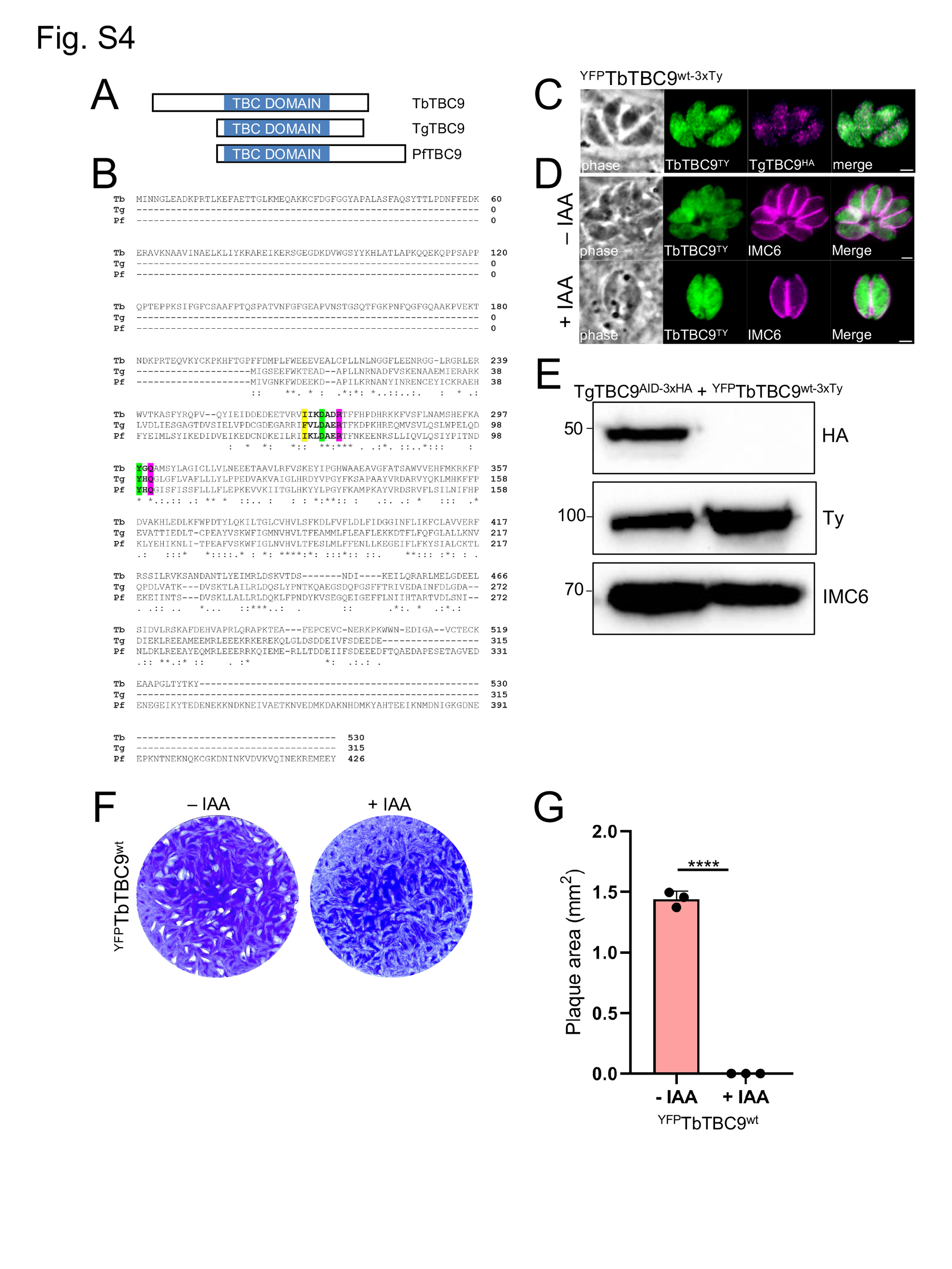

Supplement: S4 Fig — (A) Diagram showing alignment of TbTBC9, TgTBC9, and PfTBC9 revealing N-terminal extension in TbTBC9 and C-terminal extension in PfTBC9. (B) Full protein alignment of TbTBC9 (Tb), TgTBC9 (Tg), and PfTBC9 (Pf) using Clustal Omega [61]. Bold residues highlighted in yellow depict semi-conserved residues; bold residues in green depict strictly conserved residues; bold residues in magenta indicate R and Q residues important for catalytic activity. (C) IFA of YFPTbTBC9wt-3xTy colocalized with TgTBC9AID-3xHA showing overlap. Green, mouse anti-Ty; magenta, rabbit anti-HA. Scale bar = 2 μm. D) IFA of TbTBC9wt with (-) or without (+) IAA for 24 h showing that TbTBC9wt is expressed in the presence and absence of IAA. Magenta, mouse anti-Ty; green, rabbit anti-HA. Scale bar = 2 μm. (E) Western blot analysis of YFPTbTBC9wt-3xTy in the background of TgTBC9AID tagged parasites. IMC6 is a loading control. (F) Plaque assays showing that TbTBC9wt complemented parasites +IAA fail to form plaques. (G) Quantification of plaque area at day 7 showing no plaque formation by TbTBC9wt complemented parasites +IAA (****, P < 0.0001). All raw data in S1 Data. (TIF) [file pbio.3002634.s004.tif]

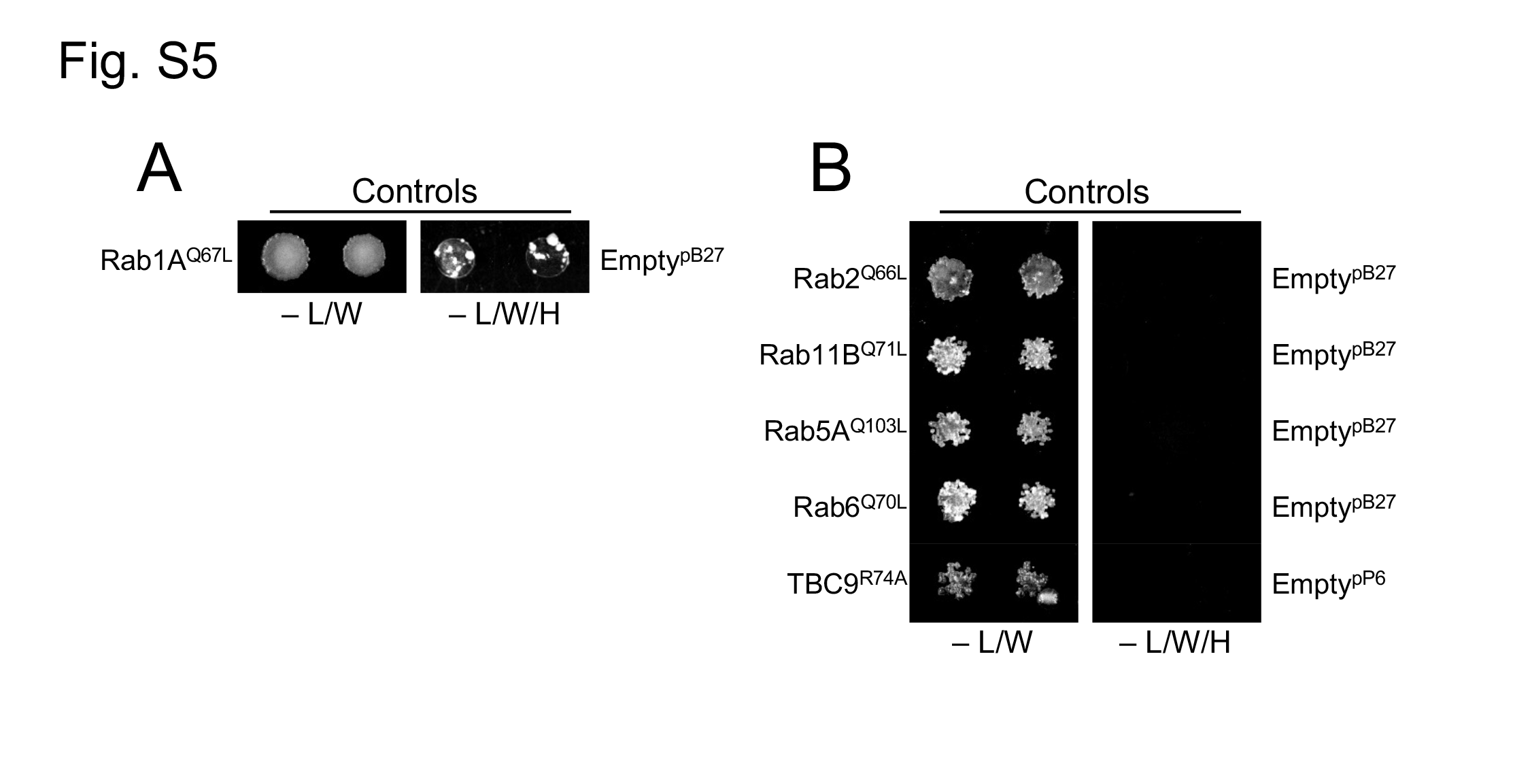

Supplement: S5 Fig — (A) Y2H of Rab1AQ67L with corresponding empty vector showing autoactivation. (B) Spot assays of pairwise Y2H demonstrates a lack of autoactivation of the indicated constructs. Each construct is coexpressed with the corresponding empty bait or prey vectors, as appropriate. (TIF) [file pbio.3002634.s005.tif]
