# Supplementary material for: Systematic characterization of all Toxoplasma gondii TBC domain-containing proteins identifies an essential regulator of Rab2 in the secretory pathway
Source: PLoS Biol. 2024 May 7;22(5):e3002634. doi: 10.1371/journal.pbio.3002634 (PMC11101121; doi:10.1371/journal.pbio.3002634)

S1_Raw_Images. Original images for blots and gels

DNA gels used in Fig 2I.
Box denotes regions used in figure, unboxed lanes are unrelated to this figure.

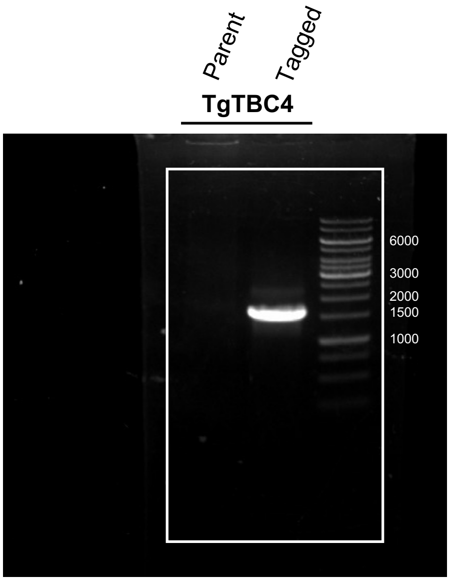

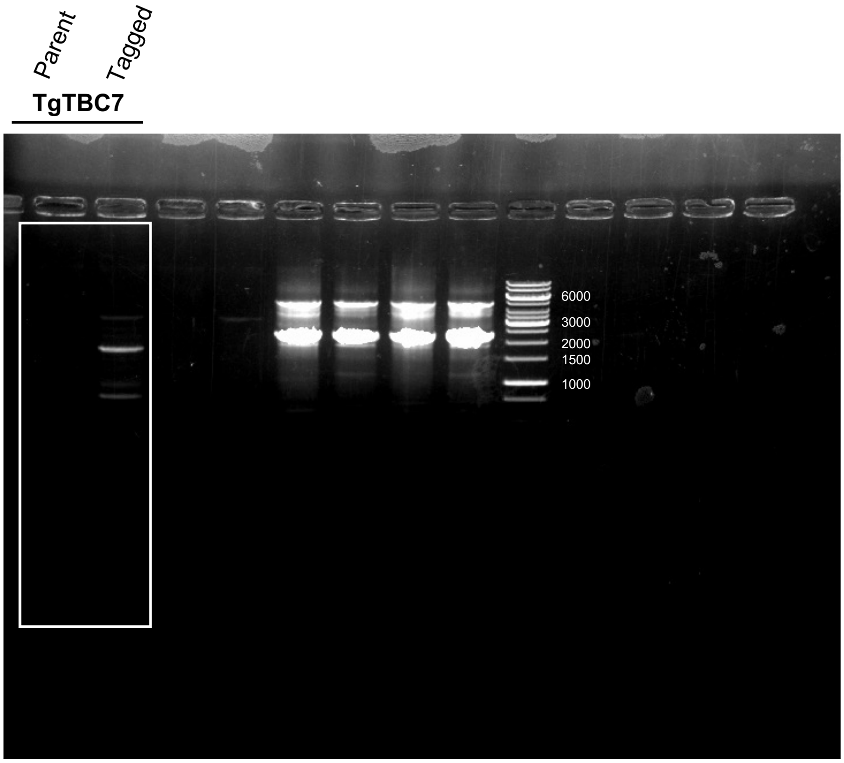

Western blot images used in Fig 3E.

Box denotes regions used in figure, unboxed lanes are unrelated to this figure.

A) Colorimetric image showing molecular weight markers

B) Chemiluminescent signal for anti-HA-HRP and anti-IMC6-HRP.

C) Merge image of A and B.

Lane numbers: 1, TgTBC9^{AID} –IAA; 2, TgTBC9^{AID} +IAA

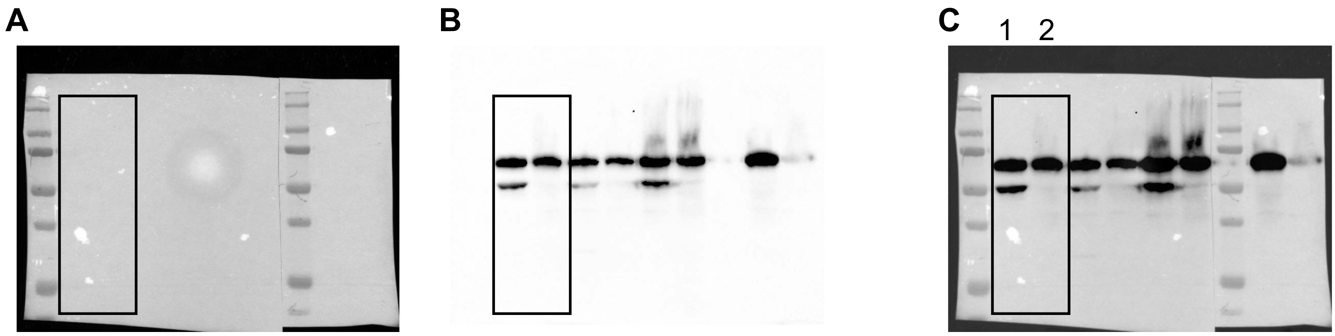

Western blot images used in Fig 5C.

Box denotes regions used in figure, unboxed lanes are unrelated to this figure.

A) Chemiluminescent signal for anti-HA-HRP and anti-IMC6-HRP.

B) Merge image of A with colorimetric image showing molecular weight markers.

C) Chemiluminescent signal for anti-Ty-HRP and anti-IMC6-HRP.

D) Merge image of C with colorimetric image showing molecular weight markers.

Lane numbers: 1, TgTBC9^{AID} –IAA; 2, TgTBC9^{AID} +IAA; 3, TgTBC9$^{wt\text{-}3xTy}$ –IAA; 4, TgTBC9$^{wt\text{-}3xTy}$ +IAA; 5, TgTBC9$^{wt\text{-}3xTy}$ –IAA; 6, TgTBC9$^{wt\text{-}3xTy}$ +IAA.

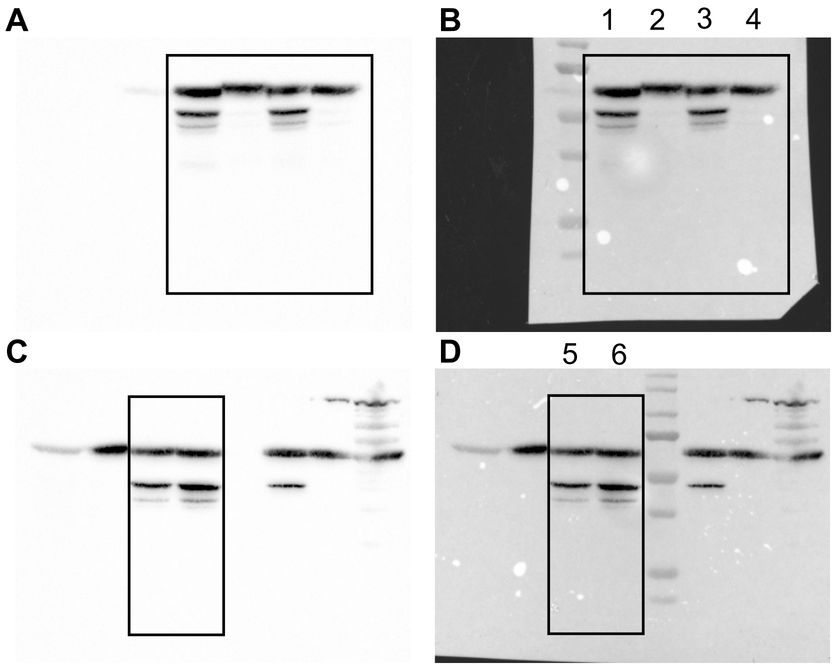

Western blot images used in Fig 5E.

A) Chemiluminescent signal for anti-Ty-HRP and anti-IMC6-HRP.

B) Merge image of A with colorimetric image showing molecular weight markers.

C) Chemiluminescent signal for anti-HA-HRP and anti-IMC6-HRP.

D) Merge image of C with colorimetric image showing molecular weight markers.

Lane numbers: 1, TBC9$^{R74A\text{-}3xTy}$ –IAA; 2, TBC9$^{R74A\text{-}3xTy}$ +IAA; 3, TBC9$^{Q101A\text{-}3xTy}$ –IAA; 4, TBC9$^{Q101A\text{-}3xTy}$ +IAA; 5, TBC9$^{R74A\text{-}3xTy}$ –IAA; 6, TBC9$^{R74A\text{-}3xTy}$ +IAA; 7, TBC9$^{Q101A\text{-}3xTy}$ –IAA; 8, TBC9$^{Q101A\text{-}3xTy}$ +IAA.

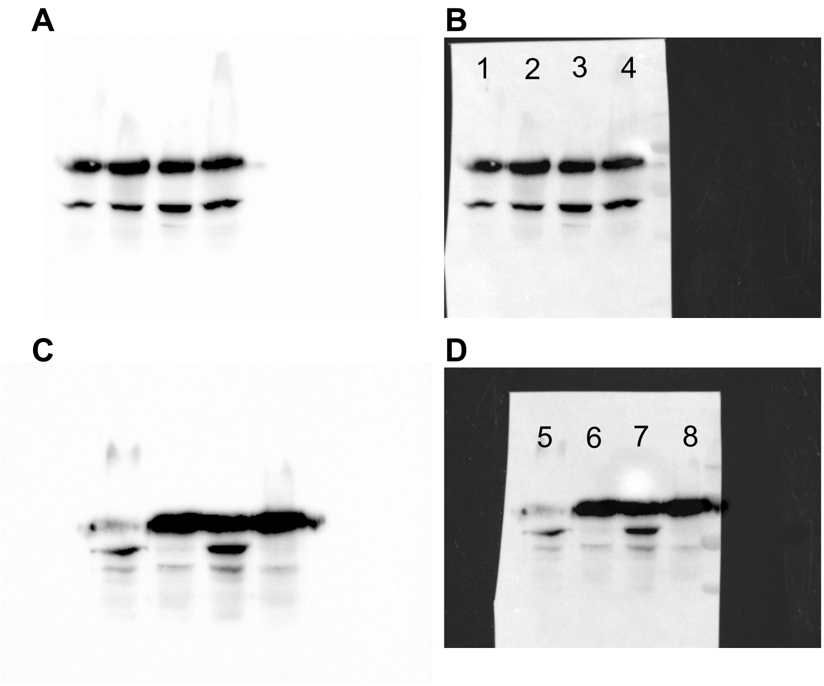

Western blot images used in Fig 6E.

Box denotes regions used in figure, unboxed lanes are unrelated to this figure.

A) Chemiluminescent signal for anti-HA-HRP and anti-IMC6-HRP.

B) Merge image of A with colorimetric image showing molecular weight markers.

C) Chemiluminescent signal for anti-Ty-HRP.

D) Merge image of C with colorimetric image showing molecular weight markers.

Lane numbers: 1, ^{YFP}PfTBC9$^{wt\text{-}3xTy}$ –IAA; 2, ^{YFP}PfTBC9$^{wt\text{-}3xTy}$ +IAA; 3, ^{YFP}PfTBC9$^{wt\text{-}3xTy}$ –IAA; 4, ^{YFP}PfTBC9$^{wt\text{-}3xTy}$ +IAA.

A

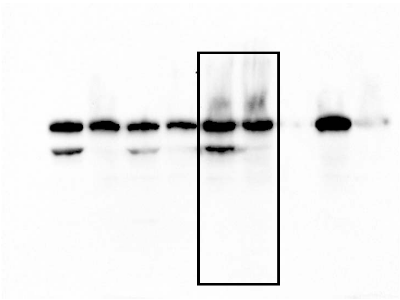

B

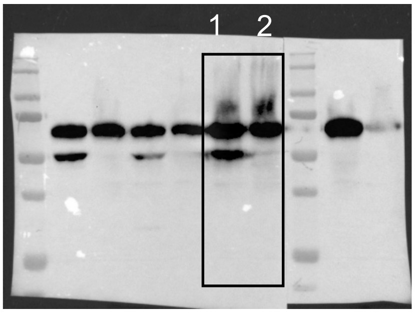

C

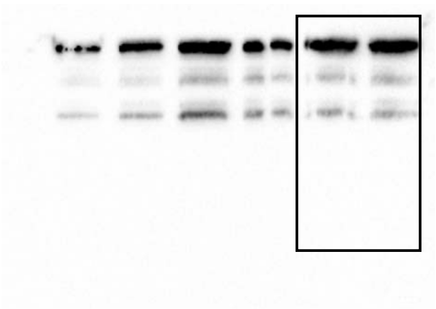

D

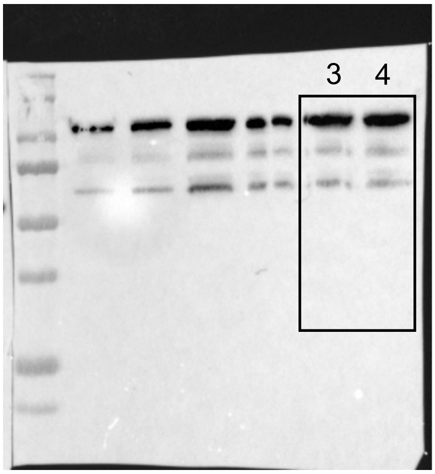

Western blot images used in Fig 6K.

Box denotes regions used in figure, unboxed lanes are unrelated to this figure.

Blots A and B were cut in half with the left half probed with anti-HA and the right half with anti-Ty.

A) Chemiluminescent signal for anti-HA-HRP.
B) Chemiluminescent signal for anti-Ty-HRP.
C) Merge image of A with colorimetric image showing molecular weight markers.
D) Merge image of B with colorimetric image showing molecular weight markers.
E) Chemiluminescent signal for anti-IMC6-HRP.
F) Merge image of C with colorimetric image showing molecular weight markers.

Lane numbers: 1, ^{YFP}TgTBC9$^{wt\text{-}3xTy}$ –IAA; 2, ^{YFP}TgTBC9$^{wt\text{-}3xTy}$ +IAA; 3, ^{YFP}TgTBC9$^{wt\text{-}3xTy}$ –IAA; 4, ^{YFP}TgTBC9$^{wt\text{-}3xTy}$ +IAA; 5, ^{YFP}TgTBC9$^{wt\text{-}3xTy}$ –IAA; 6, ^{YFP}TgTBC9$^{wt\text{-}3xTy}$ +IAA.

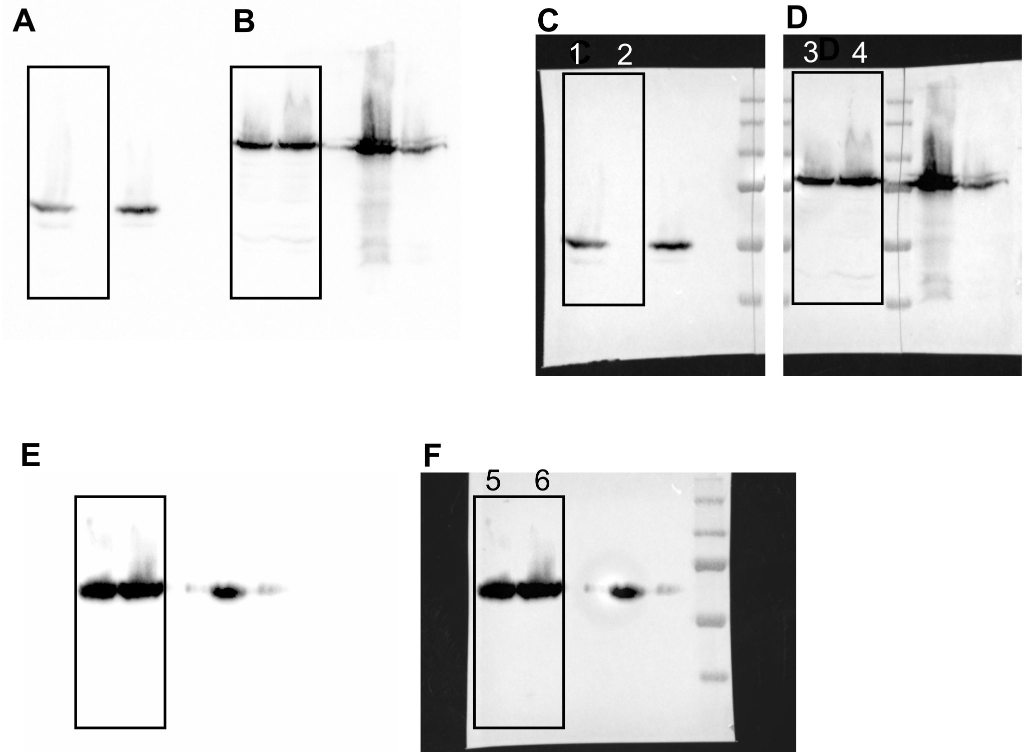

Western blot images used in Fig 7A & 7H.

Box denotes regions used in figure, unboxed lanes are unrelated to this figure.

A) Chemiluminescent signal for anti-HA-HRP.
B) Merge image of A with colorimetric image showing molecular weight markers.
C) Chemiluminescent signal for anti-V5-HRP and anti-IMC6-HRP.
D) Merge image of A with colorimetric image showing molecular weight markers.
E) Chemiluminescent signal for anti-HA-HRP.
F) Merge image of A with colorimetric image showing molecular weight markers.

Lane numbers: 1, TgTBC9^{HA} Total; 2, TgTBC9^{HA} Eluate; 3, ^{DDV5}Rab2 –IAA +Shld-1; 4, ^{DDV5}Rab2 –IAA +Shld-1; 5, TgTBC9^{AID} –IAA +Shld-1; 6, TgTBC9^{AID} +IAA +Shld-1

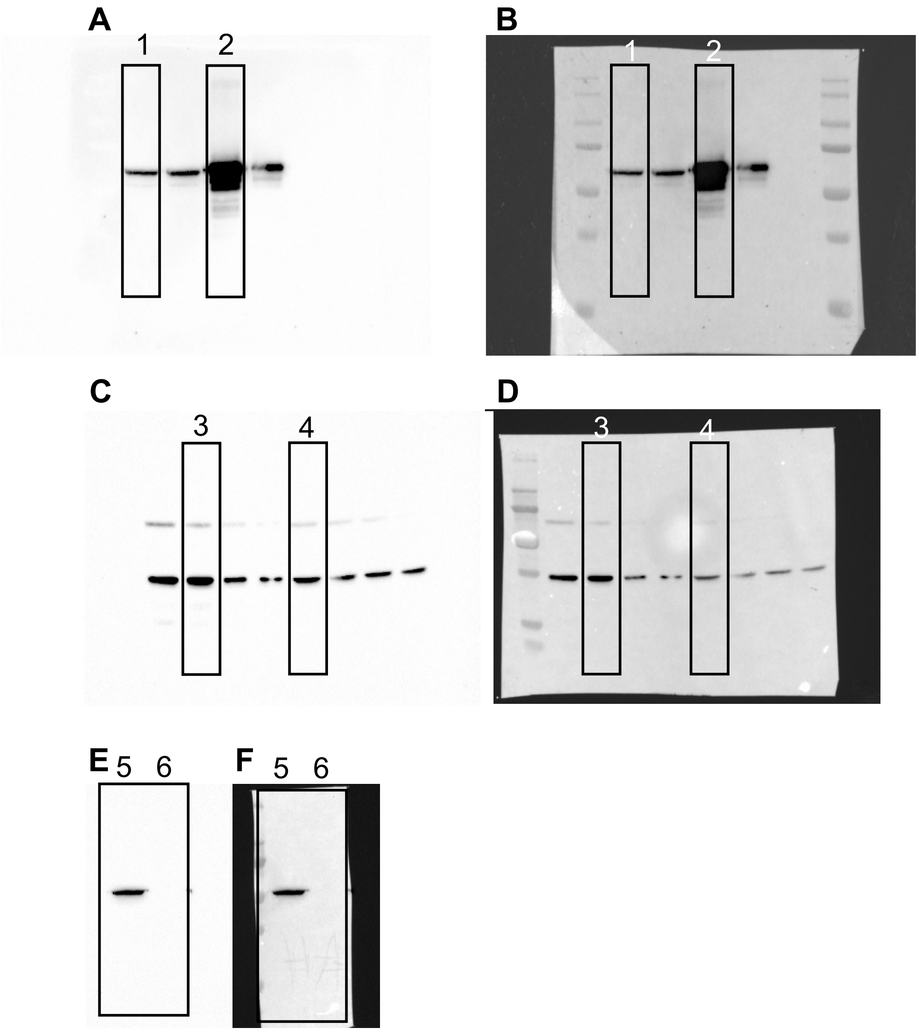

Western blot images used in Fig S2K.

Box denotes regions used in figure, unboxed lanes are unrelated to this figure.

A) Chemiluminescent signal for anti-IMC6-HRP in boxed region.

B) Chemiluminescent signal for anti-ROP7-HRP in boxed region.

C) Merge image of A and B with colorimetric image showing molecular weight markers.

Lane numbers: 1, TgTBC9^{AID} –IAA; 2, TgTBC9^{AID} +IAA, 3, TgTBC9^{AID} –IAA; 4, TgTBC9^{AID} +IAA

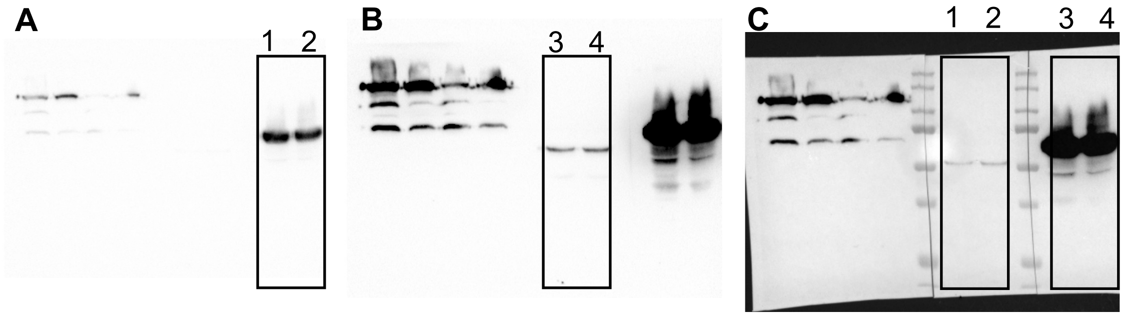

Western blot images used in Fig S2L.

Box denotes regions used in figure, unboxed lanes are unrelated to this figure.

D) Chemiluminescent signal for anti-MIC2-HRP in boxed region.

E) Chemiluminescent signal for anti-IMC6-HRP in boxed region.

F) Merge image of D and E with colorimetric image showing molecular weight markers.

Lane numbers: 1, TgTBC9^{AID} –IAA; 2, TgTBC9^{AID} +IAA, 3, TgTBC9^{AID} –IAA; 4, TgTBC9^{AID} +IAA

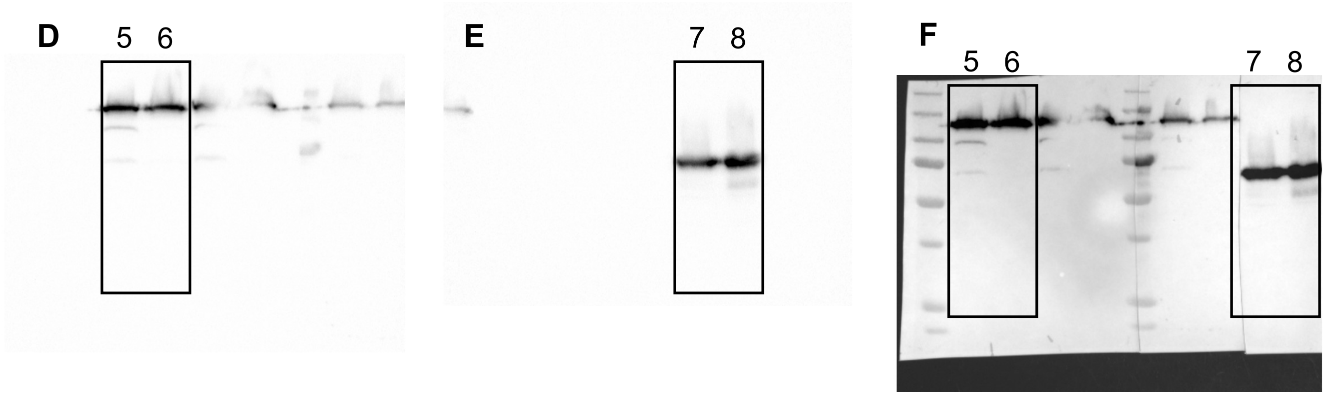

Western blot images used in Fig S4E.

Box denotes regions used in figure, unboxed lanes are unrelated to this figure.

Blots A and B were cut in half with the left half probed with anti-HA and the right half with anti-IMC6.

A) Chemiluminescent signal for anti-HA-HRP or anti-IMC6-HRP.

B) Merge image of A with colorimetric image showing molecular weight markers.

C) Chemiluminescent signal for anti-Ty-HRP.

D) Merge image of A with colorimetric image showing molecular weight markers.

Lane numbers: 1, –IAA ; 2, +IAA; 3, –IAA ; 4, +IAA; 5, –IAA ; 6, +IAA

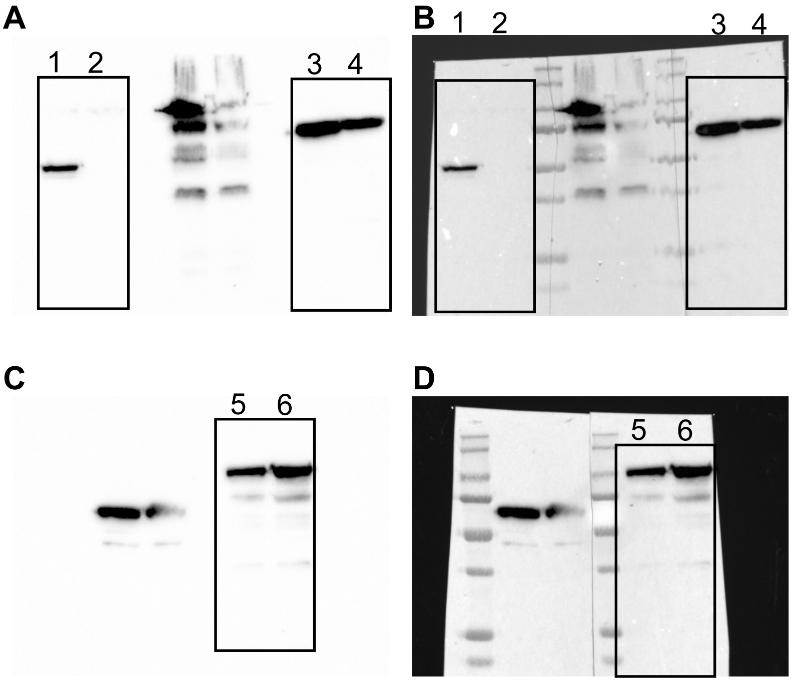

Supplement: S1 Raw Images — (PDF) [file pbio.3002634.s011.pdf]
